# Comparison of ramped and sniffing position for endotracheal intubation in obese patients: a systematic review and meta-analysis

**DOI:** 10.62675/2965-2774.20260413

**Published:** 2026-07-16

**Authors:** Gopal Krishna Basavanna, Berta Grases Garcia, Rebecca Fonseca de Azevedo, Lukács Sándor Lesinszki, Bezalel Hakkeem, Shubhangi Humbre

**Affiliations:** 1 Mysore Medical College and Research Institute Mysore Karnataka India Mysore Medical College and Research Institute - Mysore, Karnataka, India.; 2 Universitat Internacional de Catalunya Barcelona Spain Universitat Internacional de Catalunya - Barcelona, Spain.; 3 Medical University of Warsaw Warsaw Poland Medical University of Warsaw - Warsaw, Poland.; 4 Semmelweis University Budapest Hungary Semmelweis University - Budapest, Hungary.; 5 Government Medical College Kozhikode Kerela India Government Medical College - Kozhikode, Kerela, India.; 6 King Edward Memorial Hospital Pune Maharashtra India King Edward Memorial Hospital - Pune, Maharashtra, India.

**Keywords:** Airway management, Endotracheal intubation, Laryngoscopy, Obesity, Critical care, Respiration, artificial

## Abstract

**Objective:**

To evaluate the effectiveness of the ramp position over the sniffing position for endotracheal intubation in obese patients.

**Methods:**

We performed a systematic review and meta-analysis comparing ramping with sniffing position for endotracheal intubation in obese patients. We systematically searched PubMed®, Embase, and the Cochrane Library. Primary outcomes included Cormack-Lehane grading and first-pass success. We applied a random-effects model to pool relative risks and mean differences with 95% confidence intervals. Statistical analyses were performed using R 4.4.2.

**Results:**

We included four randomized controlled trials and two cohort studies. Of the 938 participants, 54.16% (508) were intubated in the ramp position. Ramping did not improve first pass success (RR 1.07; 95%CI 0.98 - 1.16; p = 0.14; I2 = 61.6%), Cormack-Lehane grades 1 - 2 (RR 0.99; 95%CI 0.97 - 1.02; p = 0.61; I2 = 0%), or Cormack-Lehane grades 3 - 4 (RR 1.94; 95%CI 0.86 - 4.37; p = 0.11; I2 = 0%). Although ramping decreased mean tube insertion and intubation time, these results did not persist in the sensitivity analyses. However, ramping decreased the number of intubation attempts in the operating room subgroup (RR 0.33; 95%CI 0.19 - 0.58; p < 0.001; I2 = 0%) and video-laryngoscopy subgroup (RR 0.33; 95%CI 0.15 - 0.70; p = 0.02; I2 = 0%).

**Conclusion:**

Our results suggest that ramping offers first-pass success and laryngeal view rates comparable to those of the sniffing position. In terms of secondary outcomes, patients in the operating room or those intubated with a video-laryngoscopy may benefit from decreased intubation attempts. Future studies with standardized definitions are required to evaluate these findings in different settings.

**PROSPERO register**: CRD42025638839

## INTRODUCTION

Recent data predict that nearly 60 percent of the adult population will be affected by obesity by 2050.^(1)^ The pathological effects of obesity on the respiratory anatomy and physiology range from decreased ventilatory capacities to rapid desaturation stemming from decreased compliance of the respiratory system as a whole, alongside increased resistance and increased metabolic rate. Most importantly, these effects are compounded by anesthesia and improper positioning.^(2)^

These factors, compounded by limited evidence and varying results on optimal positioning in obese patients, pose a challenge for airway management in this population.^(3-8)^ Guidelines recommend the use of ‘ramped’ positioning for obese patients over the traditional ‘sniffing’ position.^(9)^ The sniffing position is aimed at aligning the oral axis with the pharyngeal and laryngeal axes to facilitate intubation,^(9-11)^ which may not be possible in obese patients owing to their restricted neck flexion.^(4)^ The ramping position, defined as the elevation of head and shoulders to achieve horizontal alignment of the external auditory meatus (EAM) with the sternal notch (SN), provides a potential alternative.^(9,11,12)^

While previous meta-analyses on the general population suggest no difference between the two positions,^(13,14)^ reports on the obese population have been mixed. While some studies report improvements in first-pass success (FPS), laryngeal exposure, ease of intubation, and intubation time, others report no difference in these outcomes.^(3-7,15)^ Additionally, worsening glottic view, increased attempts, and difficulty with ramping have been reported.^(16)^ In view of this uncertainty, we designed a systematic review and meta-analysis to evaluate the effectiveness of the ramp position over the sniffing position for endotracheal intubation in obese patients.

## METHODS

This systematic review and meta-analysis were performed and reported in accordance with the Cochrane Handbook of Systematic Reviews of Interventions and the Preferred Reporting Items for Systematic Reviews and Meta-Analyses (PRISMA) guidelines.^(17,18)^ The protocol was registered in the International Prospective Register of Systematic Reviews (PROSPERO) database under registration number CRD42025638839.

### Eligibility Criteria

We included randomized controlled trials (RCTs) or observational studies; that compared ramp position with sniff position for endotracheal intubation; comprising obese patients; reported any outcomes of interest; and published in the English language. We excluded case reports, abstracts, reviews, letters to editors, systematic reviews, and meta-analyses, and studies on cadavers or mannequins.

We included studies that defined ‘ramp’ position as the horizontal alignment between the SN and EAM, and ‘sniff’ position as neck flexion and head extension at the atlantooccipital joint, irrespective of the means used to achieve these positions.

Obesity was defined as a body mass index (BMI) > 30kg/m^2^, consistent with the World Health Organization (WHO) definition.^(19)^ When such information was not available, we included studies documenting patients as ‘obese’ or weighing more than 100kgs, consistent with Emergency Department (ED) literature.^(20)^

### Search strategy and data extraction

We systematically searched PubMed, Embase, and Cochrane from inception to February 2026 using the keywords: ‘obesity’, ‘intubation’, ‘laryngoscopy’, ‘airway management’, ‘sniff’, ‘ramp’, ‘head-up’, ‘head elevated’. The full search strategy is available in table 1S (Supplementary Material). Two authors independently screened the results for inclusion and resolved discrepancies through a consensus. Additionally, we manually searched the references of the included studies and contacted the authors to request additional data for inclusion.

Three authors independently extracted data following the pre-specified search criteria. Conflicting data were evaluated by two authors and resolved through consensus.

### Endpoints and subgroup analysis

Our primary outcomes of interest were FPS and Cormack-Lehane (CL) grading of the laryngeal view,^(21)^ categorized as CL grade 1 - 2 and CL grade 3 - 4 for clinical relevance.^(22)^ Secondary outcomes included the number of intubation attempts, mean laryngoscopy time, mean tube insertion time, and mean total intubation time. The number of intubation attempts was dichotomized into those requiring > 1 attempt, based on prior research.^(23)^ Other outcomes have been defined in the supplement.

We performed a prespecified subgroup analysis of RCTs and post hoc subgroup analyses of patient setting (operating room *versus* non-operating room settings) and type of laryngoscopy (direct laryngoscopy, video laryngoscopy or both).

### Quality assessment

Two authors independently evaluated the risk of bias in randomized studies using the Cochrane Collaboration Risk of Bias assessment tool version 2 (RoB-2).^(24)^ Non-randomized studies were assessed using the Risk of Bias in Non-randomized Studies - of Interventions tool (ROBINS-I V2).^(25,26)^ Quality of evidence was assessed using the GRADE (Grading of Recommendations, Assessment, Development, and Evaluations) framework.^(27)^ Additionally, publication bias was visually inspected using funnel plots.^(28)^

### Statistical analysis

We employed a random-effects model to compute risk ratios (RR) with 95% confidence intervals (95%CI) using the Mantel-Haenszel method for binary endpoints, and mean differences (MD) with 95%CI using the Generic Inverse Variance (GIV) method for continuous endpoints. Post-hoc analyses incorporating the GIV and Hartung-Knapp-Sidik-Jonkman (HKSJ) method to calculate 95%CI were also conducted. Statistical significance was set at p < 0.05. We assessed heterogeneity with I2 statistics and the Cochran Q test.^(17)^

We considered p values less than 0.10 to be significant for subgroup differences. The leave-one-out (LOO) method was used for sensitivity analyses. All statistical analyses were performed using R version 4.2.2 (R Foundation, Vienna, Austria).

## RESULTS

### Study selection and baseline characteristics

**Figure 1:**
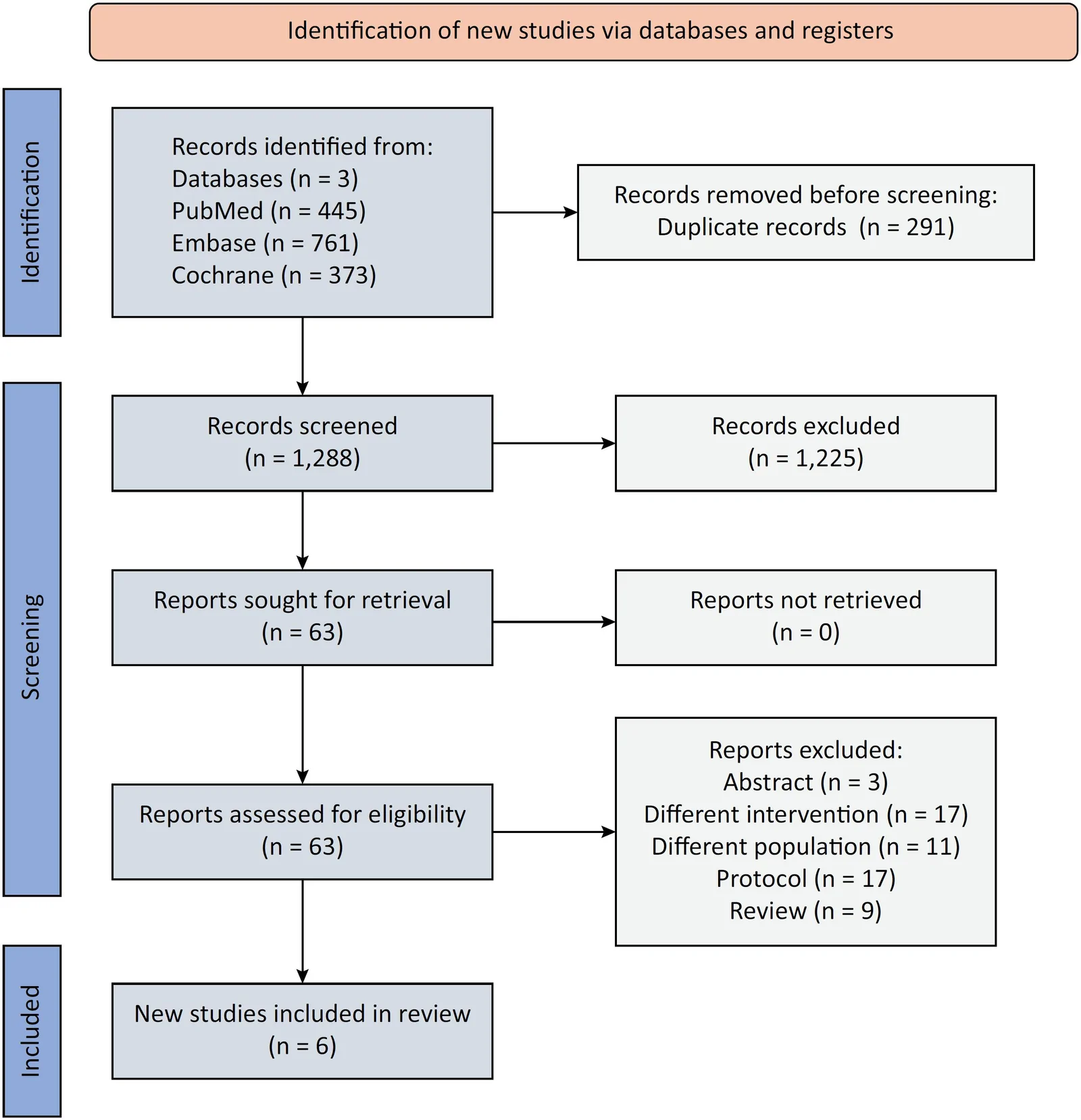
PRISMA flow diagram of study screening and study selection.

The study selection process is illustrated in figure 1. Our search strategy identified 1,579 results. After removing duplicates and screening titles/abstracts, we included 63 results for full-text review. Four RCTs and two cohort studies met our inclusion criteria.^(3-5,7,15,16)^

Individual study characteristics are provided in table 1. We included 938 patients, of whom 54.16% (508) were intubated in the ramped position. Studies achieved a ramped position using folded blankets,^(4,7,15)^ pillows,^(5)^ drapes,^(7)^ flexion of the operating room table,^(7)^ or elevation of the head of the bed,^(16)^ and the sniffing position using headrests^(4)^ or pillows.^(5,15,16)^ Four studies were undertaken in an operating room setting. Additional baseline characteristics are provided in table 2A - 2B.

**Table 1:** Characteristics of studies included in meta-analysis

Study	Design	Prespecified obesity definition	Number of patients (n)	Positioning method		Intubation characteristics			Intubator profile		
			Ramp/sniff	Ramp	Sniff	Setting	Indication	Laryngoscopy	Count	Specialty	Expertise
Bennett et al.^(3)^*	Cohort	> 100kg or if documented as obese	250/222	NA	NA	ED	Heterogeneous†	DL or VL	Multiple	ED and non-ED	Mixed‡
Collins et al.^(4)^	RCT	BMI ≥ 40kg/m^2^	33/27	Blankets	Shea headrest	OR	Elective surgery	DL	Single	Faculty Anesthesiologist	15 years
Lee et al.^(5)^	RCT	BMI ≥ 35kg/m^2^	40/41	Pillows	Kendal head positioner bagel	OR	Elective surgery	VL	Single	Faculty Anesthesiologist	10 years
Mati et al.^(7)^	RCT	BMI ≥ 30kg/m^2^	100/50	Blankets/pillows/ OR table	NA	OR	Elective surgery	VL	Multiple	Anesthesiologist	3 years
Steffi et al.^(15)^	Cohort	BMI ≥ 30kg/m^2^	40/40	Blankets	Pillow	OR	NA	VL	Multiple	Consultant anesthesiologist	5 years
Semler et al.^(16)^ *	RCT	BMI ≥ 30kg/m^2^	45/50	Elevation of the bed	Pillow	ICU	Heterogeneous§	DL or VL	Multiple	Pulmonary and critical care fellows	58 (35 - 90)¶/59 (31 - 81)||

NA - not available; ED - Emergency Department; DL - direct laryngoscopy; VL - video laryngoscopy; RCT - randomized controlled trial; BMI - body mass index; OR - operating room; ICU - intensive care unit.

* Study contains both obese and non-obese patients;

† indication of intubation included trauma, airway compromise, neurological impairment, cardiac compromise, hemodynamic compromise, overdose or ingestion, procedural, transport, tube change, chemical restraint or pain management; * indication of intubation included hypoxemic respiratory failure, hypercarbic respiratory failure, altered mental status, hemodynamic instability or preprocedural;

§ experience categorized as <10, 10-100 and >100 prior intubations;

¶ median and interquartile range of prior number of intubations in sniffing position;

|| median and interquartile range of prior number of intubations in ramping position.

**Table 2A:** Additional characteristics of studies included in meta-analysis

Study	BMI (kg/m^2^)	Age (years)	Male	ASA physical status grade (n)		Mallampati grade (n)	
	Mean ± standard deviation	Mean ± standard deviation	%	1/2/3		1/2/3	
	Intervention/control	Intervention/control	Intervention/control	Intervention	Control	Intervention	Control
Bennett et al.^(3)^*	NA	NA	NA	NA	NA	NA	NA
Collins et al.^(4)^	49.9 ± 6.9/46.9 ± 7.1	41.9 ± 8.9/43.3 ± 10.0	18.2/7.4	NA	NA	15/11/6	9/9/9
Lee et al.^(5)^	36.8 (35.6 - 40.9)/35.7 (35.2 - 38.0)†	40.7 ± 15.7/46.5 ± 14.0	47.5/48.8	0/24/16	0/21/20	24/14/3	21/20/0
Mati et al.^(7)^	41.7 ± 4.7/42.7 ± 3.8	30.7 ± 6.3/32.54 ± 6.22	57/64	29/71/ 0	11/39/0	44/51/5	22/26/2
Steffi et al.^(15)^	32.7±3.3/33±3.1	46.4 ± 13.4/50.4 ± 12.5	37.5/35	13/22/5	10/26/4	10/26/4	17/21/2
Semler et al.^(16)^*	26.7 (23.9 - 33.3)/27.3 (24.0 - 32.6)†	56(47 - 65)/56 (45 - 64)†	60.8/60.8	NA	NA	NA	NA

BMI - body mass index; ASA - American Society of Anesthesiologists; NA - not available.

* Study contains both obese and non-obese patients;

† median (interquartile range).

**Table 2B:** Additional characteristics of studies included in meta-analysis

Study	Thyromental distance (cm)	Sternomental distance (cm)	Neck circumference (cm)	Interincisor distance (cm)
	Mean ± standard deviation	Mean ± standard deviation	Mean ± standard deviation	Mean ± standard deviation
	Intervention/control	Intervention/control	Intervention/control	Intervention/control
Bennett et al.^(3)^*	NA	NA	NA	NA
Collins et al.^(4)^	11.3 ± 1.0/11.1 ± 1.2	16.7 ± 1.5/16.7 ± 1.5	47.9 ± 5.3/43.4 ± 6.7	4.8 ± 0.6/4.4 ± 0.5
Lee et al.^(5)^	8.1 (6.8 - 9.0)/8.2 (7.0 - 8.8)†	16.0(14.9 - 17.0) /15.2 (14.5 - 16.8)†	45.1 ± 5.6/45.2 ± 5.0	4.9 (4.5 - 5.0)/5.0 (4.4 - 5.2)†
Mati et al.^(7)^	8.3 ± 1.3/8.3 ± 1.2	NA	41.1 ± 1.6/41.8 ± 1.5	NA
Steffi et al.^(15)^	NA	NA	NA	NA
Semler et al.^(16)^*	NA	NA	NA	NA

NA - not available.

* Study contains both obese and non-obese patients;

† median (interquartile range).

### Pooled analysis of primary outcomes

**Figure 2:**
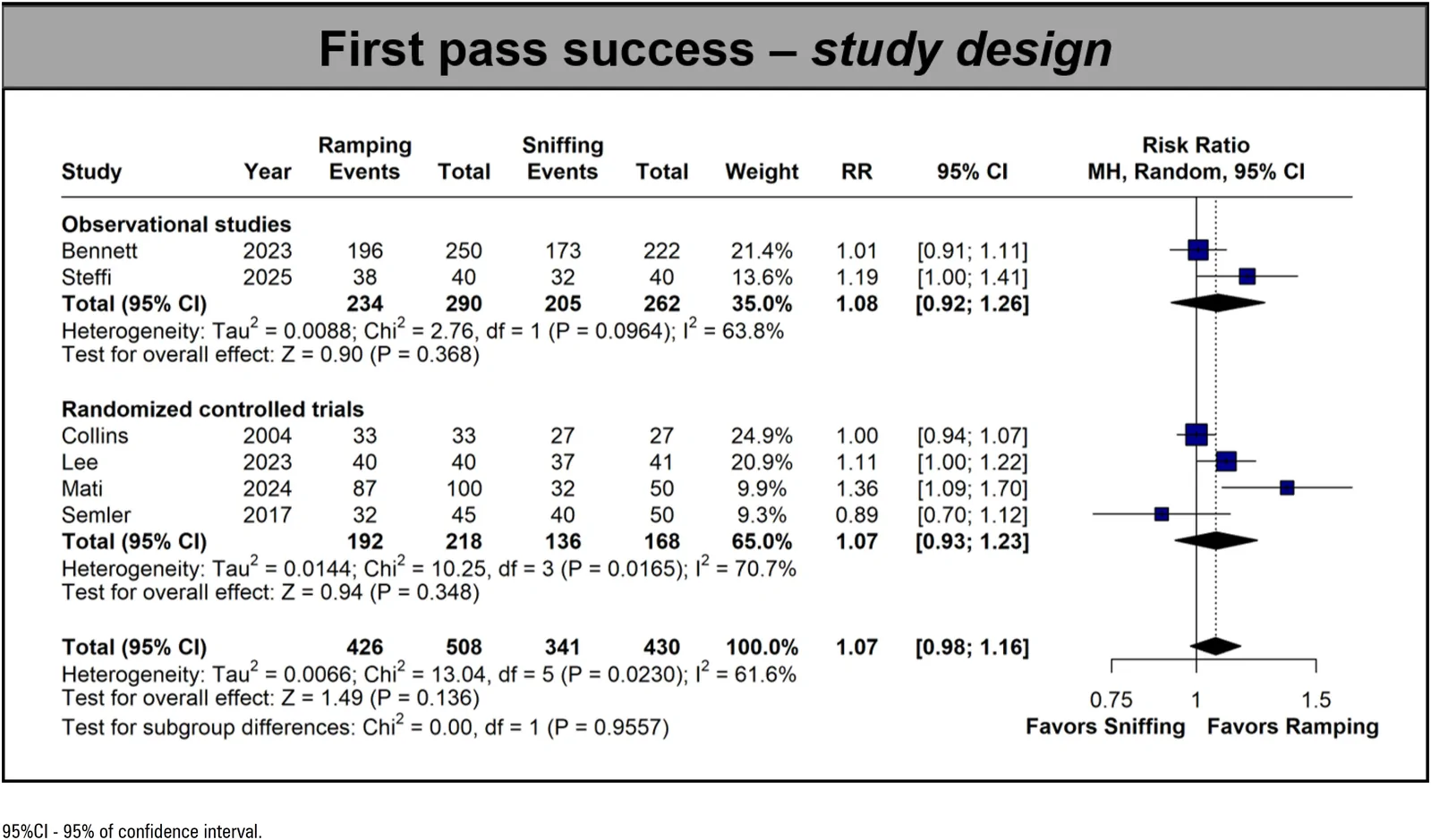
Random effects meta-analysis of first pass success stratified by study design type.

Six studies reported FPS rates. The ramp position did not improve FPS (RR 1.07; 95%CI 0.98 - 1.16; p = 0.14; I2 = 61.6%; Figure 2). Although a prespecified subgroup analysis of RCTs (p = 0.95, Figure 2) did not reveal any differences, post-hoc analyses sub-grouped by patient setting (p = 0.09, Figure 1SA [Supplementary Material]) and type of laryngoscopy (p = 0.02, Figure 1SB [Supplementary Material]) revealed significant subgroup differences. The results remained stable in the LOO analysis, with Mati et al.,^(7)^ being the major contributor to heterogeneity (Figure 1SC - Supplementary Material).

**Figure 3:**
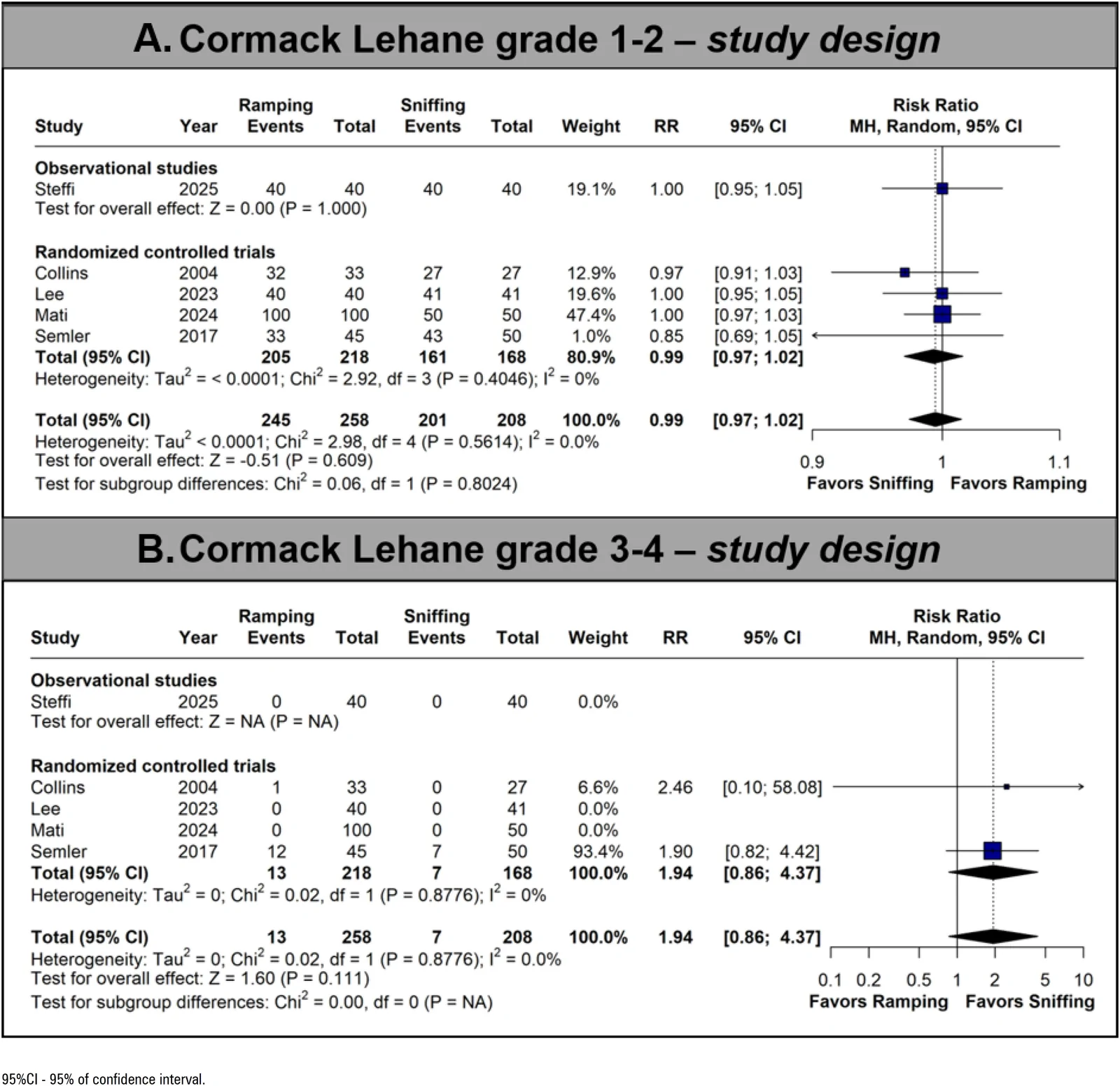
Random effects meta-analysis of (A) Cormack-Lehane grades 1 - 2 and (B) Cormack-Lehane grades 3 - 4 stratified by study design.

Five studies reported CL grading. Pooled analysis revealed no difference in CL grade 1 - 2 (RR 0.99; 95%CI 0.97 - 1.02; p = 0.61; I2 = 0%; Figure 3) or CL grade 3 - 4 (RR 1.94; 95%CI 0.86 - 4.37; p = 0.11; I2 = 0%; Figure 3). The result remained stable during the LOO analysis (CL 1 - 2 [Figure 2SA - Supplementary Material]; CL 3 - 4 [Figure 2SB - Supplementary Material]), and post-hoc analyses of patient setting (CL 1 - 2, p = 0.15, [Figure 2SC - Supplementary Material]; CL 3 - 4, p = 0.88, [Figure 2SD - Supplementary Material]) or different laryngoscopy subgroups (CL 1 - 2, p = 0.51, [Figure 2SE - Supplementary Material]; CL 3 - 4, p = 0.88, [Figure 2SF - Supplementary Material]) also failed to reveal any differences.

Additionally, post-hoc analyses incorporating the GIV and HKSJ methods, conducted in view of non-rare events and heterogeneity, did not show any difference in our primary outcomes (Figure 3SA - 3SF - Supplementary Material).

### Pooled analysis of secondary outcomes

**Figure 4:**
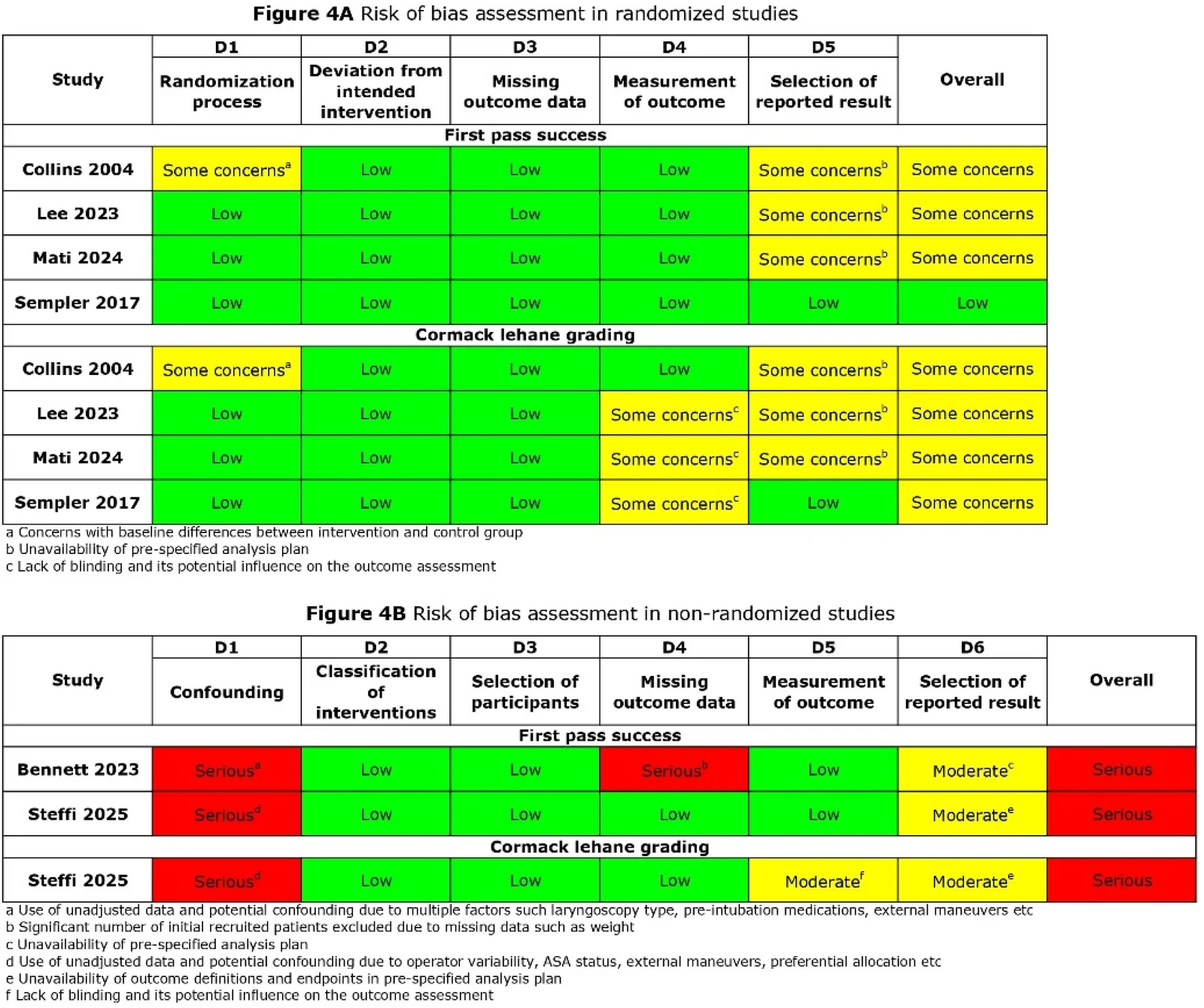
Risk of bias assessment for the included randomized and non-randomized studies.

Six studies reported the number of intubation attempts. The positions did not differ with respect to intubations requiring more than one attempt (RR 0.61; 95%CI 0.29 - 1.28; p = 0.19; I2 = 71.8%; Figure 4SA - Supplementary Material). While a subgroup analysis of RCTs revealed no difference (p = 0.39, Figure 4SA - Supplementary Material), a post-hoc analysis revealed statistically significant subgroup differences based on the patient setting (p = 0.0004, Figure 4SB - Supplementary Material) and type of laryngoscopy (p < 0.0001, Figure 4SC - Supplementary Material). Additionally, the operating room subgroup was less likely to require more than one intubation attempt in the ramp position (RR 0.33; 95%CI 0.19 - 0.58; p < 0.001; I2 = 0%; Figure 4SB - Supplementary Material), similar to the VL subgroup (RR 0.33; 95%CI 0.15 - 0.70; p = 0.02; I2 = 0%; Figure 4SC - Supplementary Material). The results remained consistent across the LOO analysis (Figure 4SD - Supplementary Material).

Three studies reported mean laryngoscopy time, which did not differ between the two positions in the pooled analysis (MD = -6.24 seconds; 95%CI = -13.71 - 1.24 seconds; p = 0.10; I2 = 98.1%; Figure 5SA - Supplementary Material). Given the low reliability of accurate confidence interval estimation in analyses with a small number of studies, we conducted a sensitivity analysis using the HKSJ method, which also revealed similar results (MD -6.24 seconds; 95%CI -22.64 - 10.16 seconds; p = 0.24; I2 = 98.1%; Figure 5SB - Supplementary Material).

Two studies reported the mean tube insertion time. Although ramping significantly decreased this interval (MD -12.22 seconds; 95%CI -18.79 - -5.56 seconds; p < 0.01; I2 = 71.3%; Figure 5SC - Supplementary Material), the effect did not persist in the HKSJ method sensitivity analysis (MD -12.22 seconds; 95%CI -54.81 - 30.37 seconds; p = 0.17; I2 = 71.3%; Figure 5SD - Supplementary Material).

Three studies reported mean intubation time with similar definitions. A pooled analysis showed that ramping significantly reduced intubation time, albeit with substantial heterogeneity (MD -11.73 seconds; 95%CI -23.16 - -0.30 seconds; p = 0.04; I2 = 96.6%; Figure 5SE - Supplementary Material). However, this result did not persist in the HKSJ method sensitivity analysis (MD -11.73 seconds; 95%CI -36.56 - 13.10 seconds; p = 0.18; I2 = 96.6%; Figure 5SF - Supplementary Material).

### Quality assessment

Risk of bias assessment for the primary outcomes are provided in figure 4. Overall, the majority of the RCTs had ‘some concerns’ of bias due to lack of blinding and unavailability of a protocol.^(24)^ The cohort studies had a ‘serious’ risk of bias due to confounding, missing data, lack of blinding, and lack of protocol.^(25)^ Risk of bias assessments for secondary outcomes are available in the figures 6SA - 6SK (Supplementary Material).

The summary of findings is presented in the supplement (Tables 2S and 3S - Supplementary Material). Certainty of the evidence among RCTs for first-pass success was very low, downgraded due to risk of bias, inconsistency, and publication bias. Similarly, certainty of evidence for CL grade 1 - 2 and CL grade 3 - 4 was low and very low, respectively, and was downgraded due to risk of bias, imprecision, and publication bias. The assessment of secondary outcomes is available in tables 2S and 3S (Supplementary Material). Although visual inspection of the funnel plots for evidence of publication bias did not suggest small-study effects (Figure 7SA - 7SC - Supplementary Material), the limited number of studies precluded a reliable assessment of publication bias.

## DISCUSSION

### Summary of key findings

In this meta-analysis of four RCTs and two cohort studies comprising 938 obese patients undergoing tracheal intubation in the ramp or sniffing position, the results showed that FPS and CL grading did not differ between the two positions; patients in the operating room and patients intubated with a VL were less likely to require more than one intubation attempt in the ramp position; and mean laryngoscopy time, mean tube insertion time, and mean intubation time did not differ between the two positions.

### Interpretation and implications for future research

Although we did not retrieve any records of a meta-analysis comparing the ramping and sniffing positions in obese patients alone, Tsan et al. and Okada et al. conducted meta-analyses on the general population, including at least one study with obese patients. Our results are similar to those reported here.^(13,14)^

The association between repeated intubation attempts with adverse complications and poor laryngeal view with difficult intubation has been well established.^(22,23,29)^ Lee et al. and Mati et al. reported increased FPS with ramping in obese patients undergoing elective surgery.^(5,7)^ In contrast, Bennet et al. reported an increased odds of FPS with ramping in the ED (OR 1.7; 95%CI 1.2 - 2.4; p = 0.002), and Semler et al. reported decreased FPS in the intensive care unit (ICU).^(3,16)^ However, we did not find any difference in our meta-analysis. This may be due to baseline differences in morbidity and suboptimal intubating conditions outside the operating room.^(30)^ Additionally, Lee et al.^(5)^ and Mati et al.^(7)^ used a VL and employed faculty anesthesiologists with considerable experience, which could explain the improved FPS.^(31-33)^ In contrast, Bennet et al.^(3)^ employed intubators with varying experience and specialties, whereas Semler et al.^(16)^ utilized trainees. Furthermore, these studies included only 18.4% and 36.5% of obese patients in their total sample sizes, respectively. Moreover, we included unadjusted data from Steffi et al.^(15)^ and Bennet et al.^(3)^ – the latter defining obesity as weight > 100kg or patients documented as ‘obese’ rather than by BMI, which could have introduced bias. To address this, we performed sensitivity analyses (Figure 1SC - Supplementary Material) and subgroup analyses (Figure 2) but failed to detect any difference. Previous meta-analyses and a recent study on supraglottic airway device (SAD) insertion in obese patients agree with our findings.^(13,14,34)^ However, this finding should be interpreted with caution due to the high degree of heterogeneity. While heterogeneity is poorly estimated in meta-analyses with few studies, a sensitivity analysis revealed that Mati et al.^(7)^ was a major contributor to heterogeneity (Figure 1SC - Supplementary Material). This could be due to the influence of VL and experienced anesthesiologists, compounded by an imbalance in sample sizes between the intervention and control arms.^(17,31)^ Additionally, while the operating room setting and VL subgroups revealed significant subgroup differences, unexplained heterogeneity within each subgroup limits the certainty of such analyses.

We found no difference in CL grades, consistent with prior meta-analyses.^(13,14)^ Although Tsan et al. observed that ramping improved glottic views in the operating room, obese patients made up only 9.5% of this subgroup.^(13)^ Additionally, Lim et al. evaluated glottic views through an SAD using a bronchoscope in obese patients and found no difference between the two positions.^(35)^ In contrast, a surgical cohort study with 56.8% of patients with BMI > 30kg/m^2^ had better glottic views in the ramped position.^(6)^ However, both these studies carried out intubation in both positions on the same patient, which may be a source of bias. Moreover, the conventional CL grading system has recently been challenged. First, given the relative rarity of grade 3 and grade 4, and the fact that even grade 1 or 2 glottic views sometimes require additional interventions, Yentis et al. suggested the use of a modified version with grade 2 subdivided into grade 2A and 2B, due to its ability to transmit more information, especially with small study samples.^(36)^ Second, O’Loughlin et al. found a higher degree of accuracy and inter-rater reliability with the Percentage of Glottic Opening (POGO) scores and the Fremantle scores over the CL grading system to document VL.^(37)^ Finally, a VL could uncouple glottic view and intubation difficulty, thus raising uncertainties over the continued applicability of the CL grading system.^(38)^

Decreased attempts and faster intubation could prove valuable in preventing rapid desaturation in patients undergoing induction, a common complication among obese patients.^(2)^ While we observed no difference in intubations requiring more than one attempt, a post-hoc subgroup analysis revealed patient setting (p = 0.0004, Figure 4SB - Supplementary Material) and type of laryngoscopy (p < 0.0001, Figure 4SC - Supplementary Material) significantly modified the effect of these positions. Additionally, the considerable reduction in heterogeneity within each subgroup and the sustained results across sensitivity analyses support the favorable outcomes of the ramp position in the operating room patients and in those intubated with a VL.

While prior meta-analyses did not report on mean laryngoscopy time or mean tube insertion time, they reported no difference in mean total intubation time.^(13,14)^ In contrast, although our study found that ramping significantly decreased mean tube insertion time (Figure 5SC - Supplementary Material) and mean intubation time (Figure 5SE - Supplementary Material), this result did not persist in the sensitivity analyses (Figure 5SD, Figure 5SF - Supplementary Material). Hence, while drawing rigid conclusions from these two outcomes is limited by the high heterogeneity and the small number of studies, it is worth noting that the data come entirely from patients in the operating room.

We identified only a few studies that reported other relevant outcomes, including ease of intubation, difficulty with mask ventilation, use of ancillary maneuvers or adjuncts, and adverse events. Lee et al. observed that ramping significantly increased the percentage of easy intubation (70.0% *versus* 7.3%; p < 0.001), as measured by the intubation difficulty scale.^(5)^ Similarly, Mati et al. reported a decrease in difficulty in the 25º back-up position, while Semler et al. reported an increase in difficulty in the ramped position.^(7,16)^ However, we were not able to pool these outcomes due to the different scales used. Furthermore, the American Society of Anesthesiologists’ definition of a ‘difficult airway’ also involves ‘difficulty in mask ventilation’, an outcome less frequently reported in trials.^(39)^ Lee et al. observed a decreased difficulty in mask ventilation with ramping (2.5% *versus* 34.1%; p < 0.001).^(5)^

Prior studies have reported an increase in the safe-apnea period in the ramp position. However, this outcome has rarely been evaluated outside the operating room.^(12,40,41)^ ICU trials assess a surrogate outcome - the lowest oxygen saturation, which did not differ between the two positions.^(16)^ In contrast, a study in the ED observed 31.1% of adverse events associated with ramping, and 24.5% with sniffing.^(3)^ Since these studies included only 36.5% and 18.4% of obese patients, respectively, future studies examining adverse events in different settings will be necessary.

### Limitations

This study has some limitations. First, there was considerable heterogeneity in our review due to varying definitions and methodologies. Although we attempted to address this limitation using sensitivity and subgroup analyses, the use of advanced statistical methods, such as meta-regression, was not possible due to the small number of studies. Second, the effect of ramping on obese patients outside the operating room is unclear due to a limited sample size. We included one study in the ED and one study in the ICU, and both these studies included only 18.4% and 36.5% of obese patients in their study population, respectively. Finally, we included unadjusted data from cohort studies, and most RCTs lacked blinding, which could have biased our analysis.

## CONCLUSION

In this meta-analysis comparing the ramping and sniffing positions for endotracheal intubation in obese patients, we observed that ramping achieved comparable first-pass success and laryngeal view rates to the sniffing position. While ramping significantly decreased the number of intubation attempts in a subgroup of patients in the operating room and among those intubated with video laryngoscopy, it did not result in faster intubation times. Future studies with standardized definitions are needed to evaluate these findings in different settings.

## Data Availability

The datasets analyzed during the current study were obtained from previously published studies cited in the reference list. The data extraction forms and statistical analysis files are available from the corresponding author upon reasonable request.
