# Supplementary material for: Comparison of ramped and sniffing position for endotracheal intubation in obese patients: a systematic review and meta-analysis
Source: Crit Care Sci. 2026 Jul 16;38:e20260413. doi: 10.62675/2965-2774.20260413 (PMC13518020; doi:10.62675/2965-2774.20260413)
Supplement: Supplementary file 1 [file 2965-2774-ccsci-38-e20260413-Suppl01.pdf]

Critical Care Science
https://doi.org/10.62675/2965-2774.20260413

SUPPLEMENTARY MATERIAL

Comparison of ramped and sniffing position for endotracheal intubation in obese patients: a systematic review and meta-analysis

Gopal Krishna Basavanna[1], Berta Grases Garcia[2], Rebecca Fonseca de Azevedo[3], Lukács Sándor Lesinszki[4], Bezalel Hakkeem[5], Shubhangi Humbre[6]

Table 1S - Search strategy

Database	Search Strategy
PubMed®	(Obese OR Obesity) AND (Intubation OR Laryngoscopy OR "Airway Management" OR "Airway Management"[MeSH]) AND (Position OR Sniff* OR Ramp* OR "head up" OR "head elevated")
Embase	(Obese OR Obesity) AND (Intubation OR Laryngoscopy OR "Airway Management") AND (Position OR Sniff* OR Ramp* OR "head up" OR "head elevated")
Cochrane Library	(Obese OR Obesity) AND (Intubation OR Laryngoscopy OR "Airway Management") AND (Position OR Sniff* OR Ramp* OR "head up" OR "head elevated")

DEFINITION OF SECONDARY OUTCOMES

1. Mean laryngoscopy time

Time interval between the insertion of the laryngoscope into the patient's mouth till the best glottic view was achieved.

2. Mean tube insertion time

Time interval between the insertion of the endotracheal tube into the patient's mouth until the passage of the tube through the glottis through the glottis.

3. Mean intubation time

Time interval between the insertion of endotracheal tube into the patient's mouth till the passage of the tube through the into the trachea.

This is an open access article under the CC BY license (https://creativecommons.org/licenses/by/4.0/).

PRIMARY OUTCOMES

First pass success

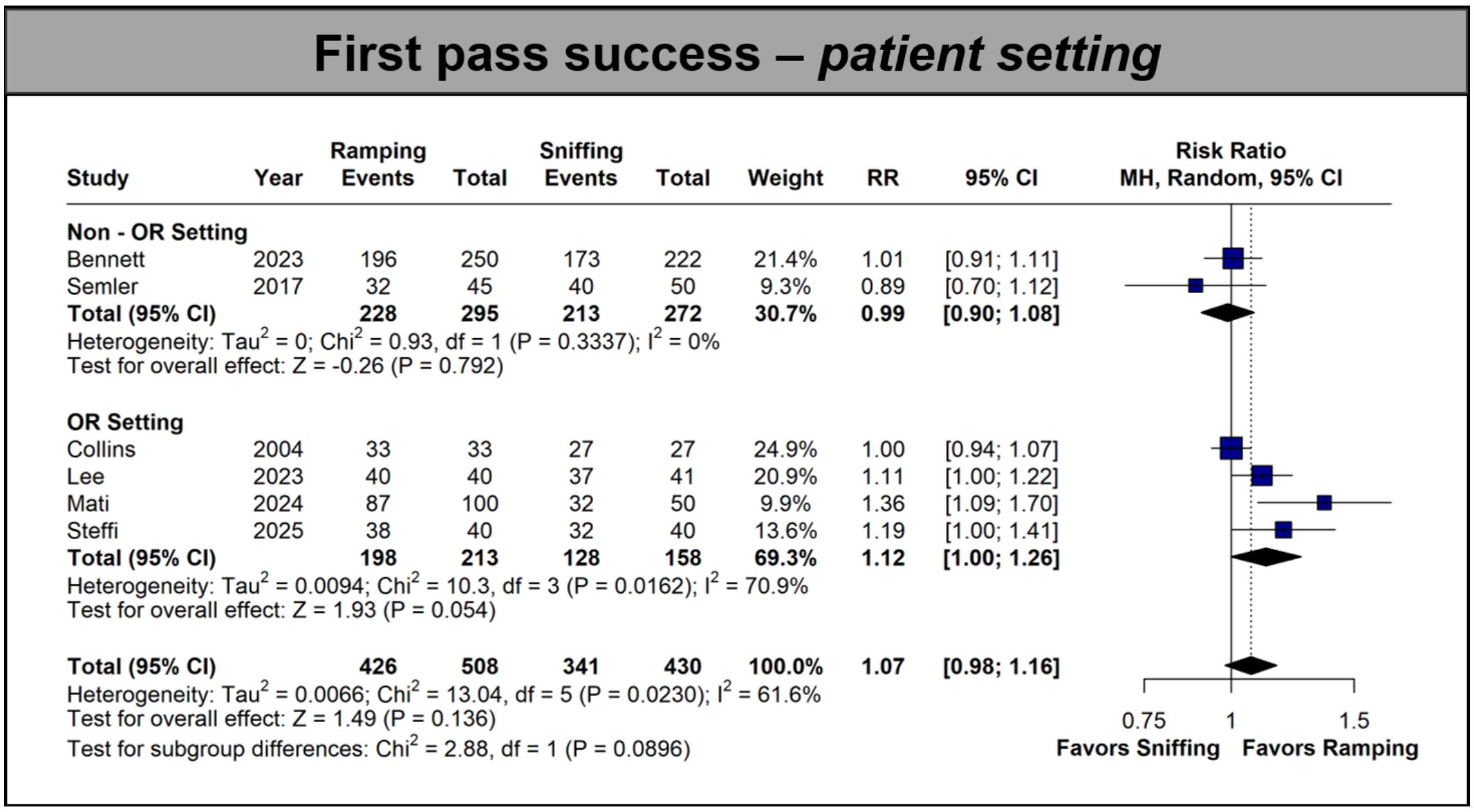

Figure 1SA - Random effects meta-analysis of first pass success stratified by patient setting.

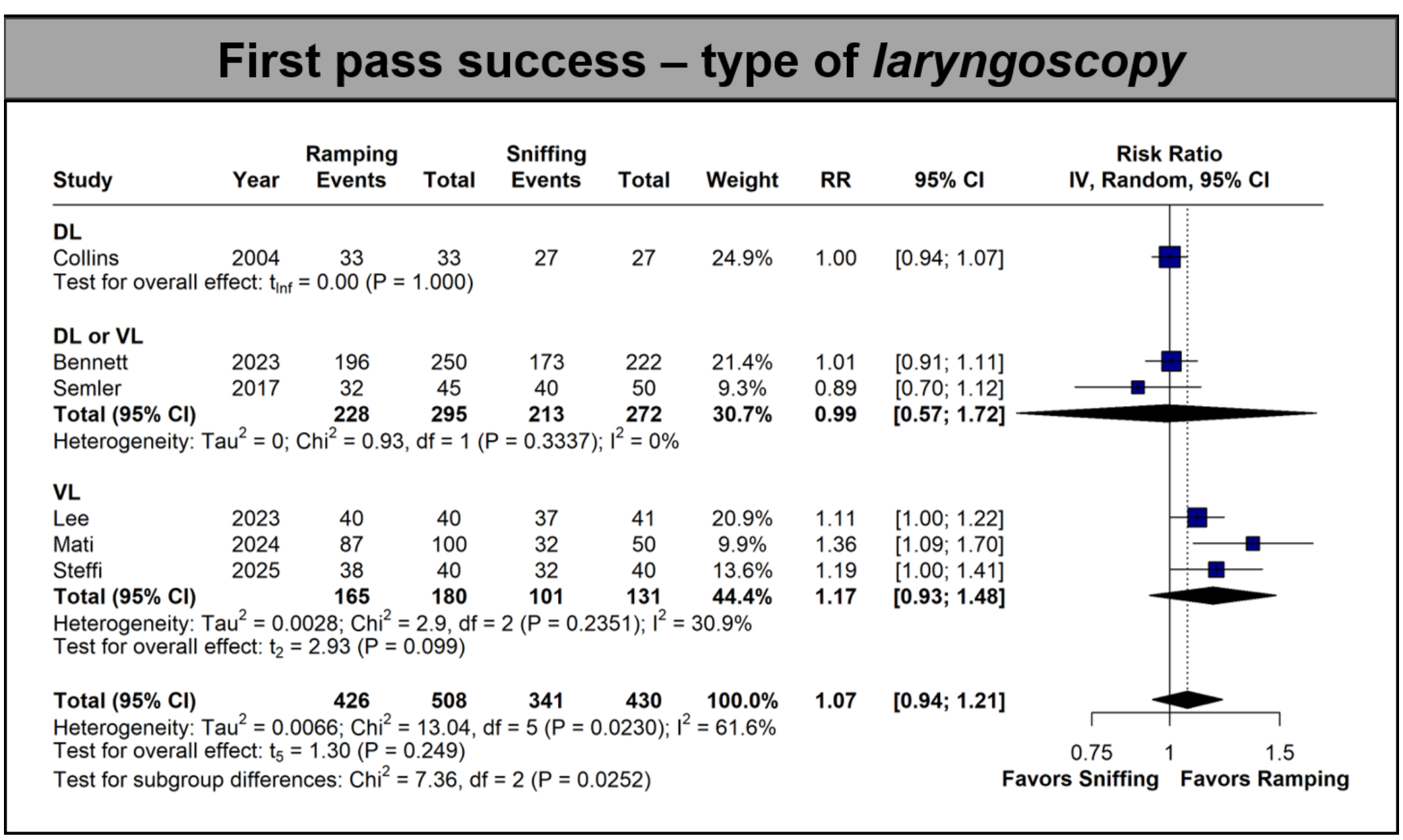

Figure 1SB - Random effects meta-analysis of first pass success stratified by type of laryngoscopy.

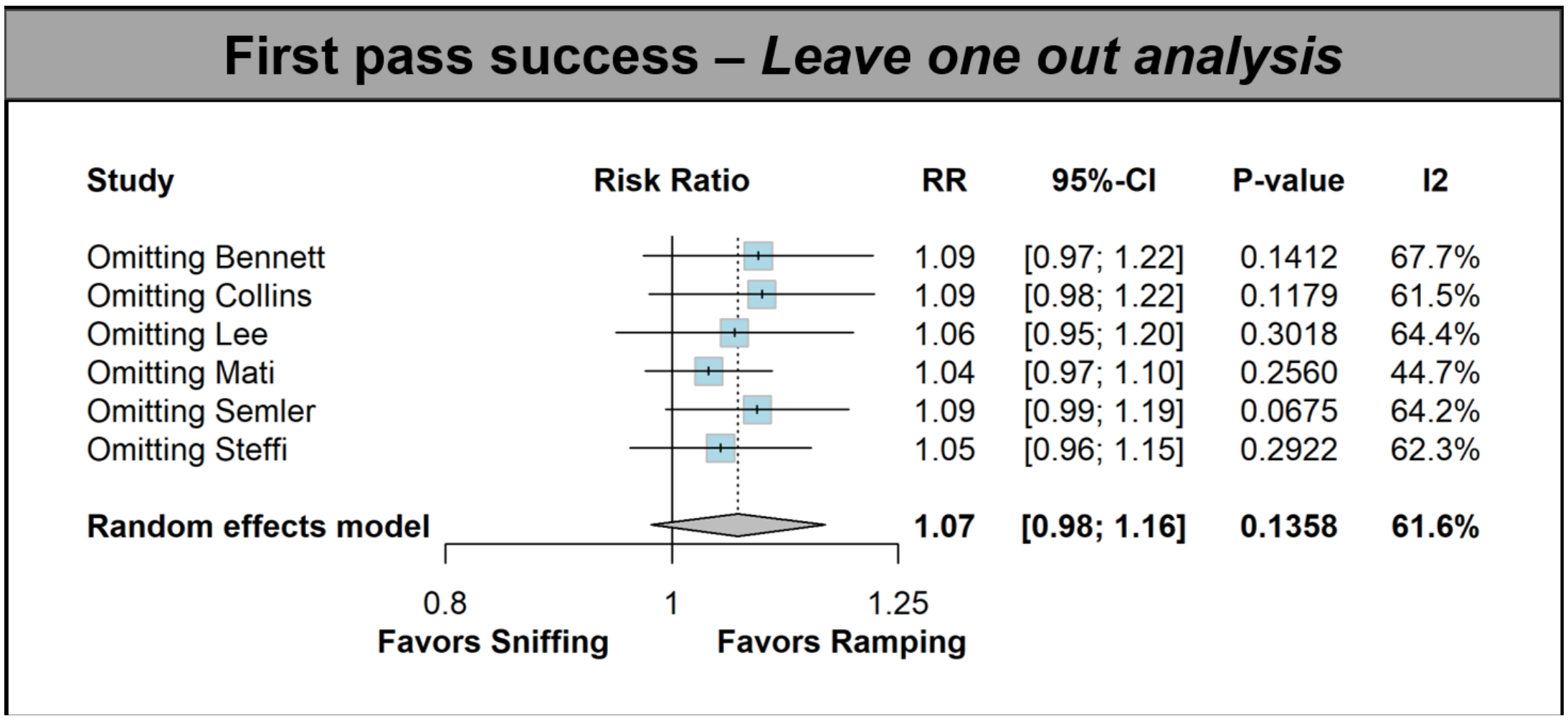

Figure 1SC - Leave one out sensitivity analysis of first pass success.

CORMACK-LEHANE GRADING

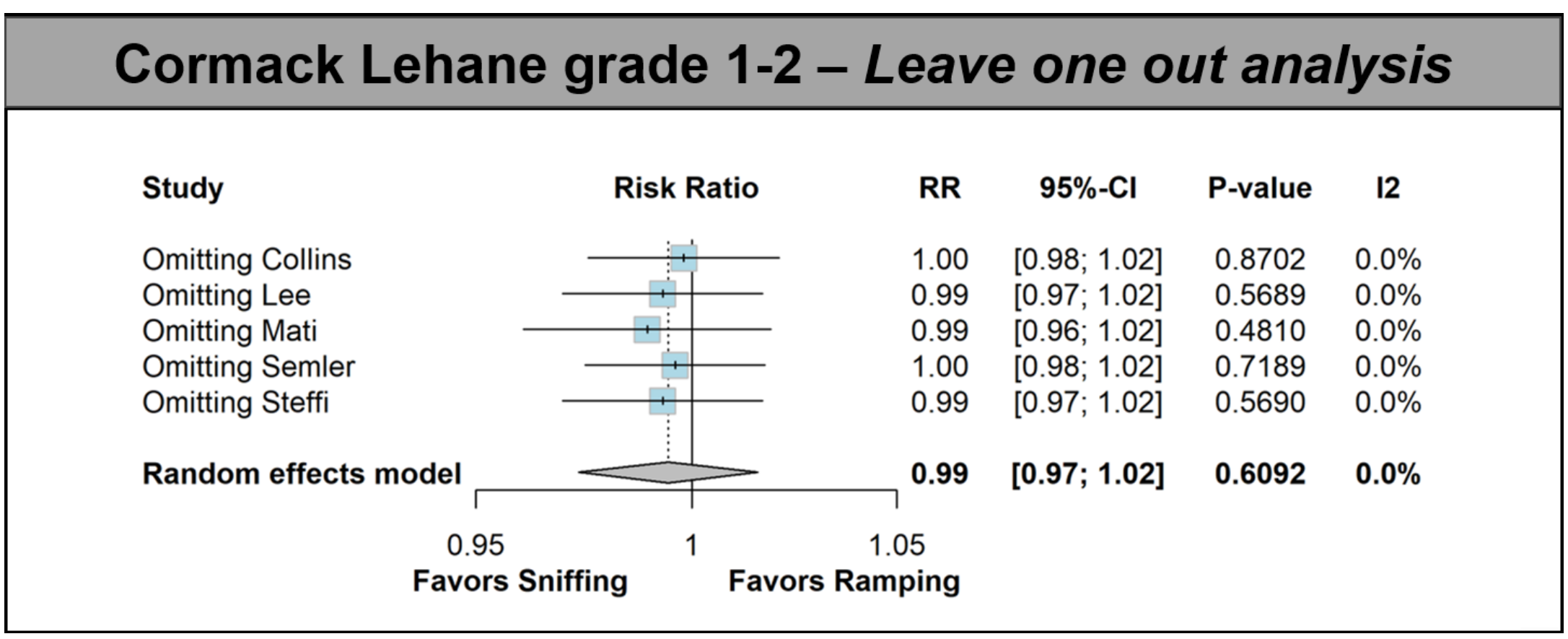

Figure 2SA - Leave one out sensitivity analysis of Cormack-Lehane grade 1 - 2.

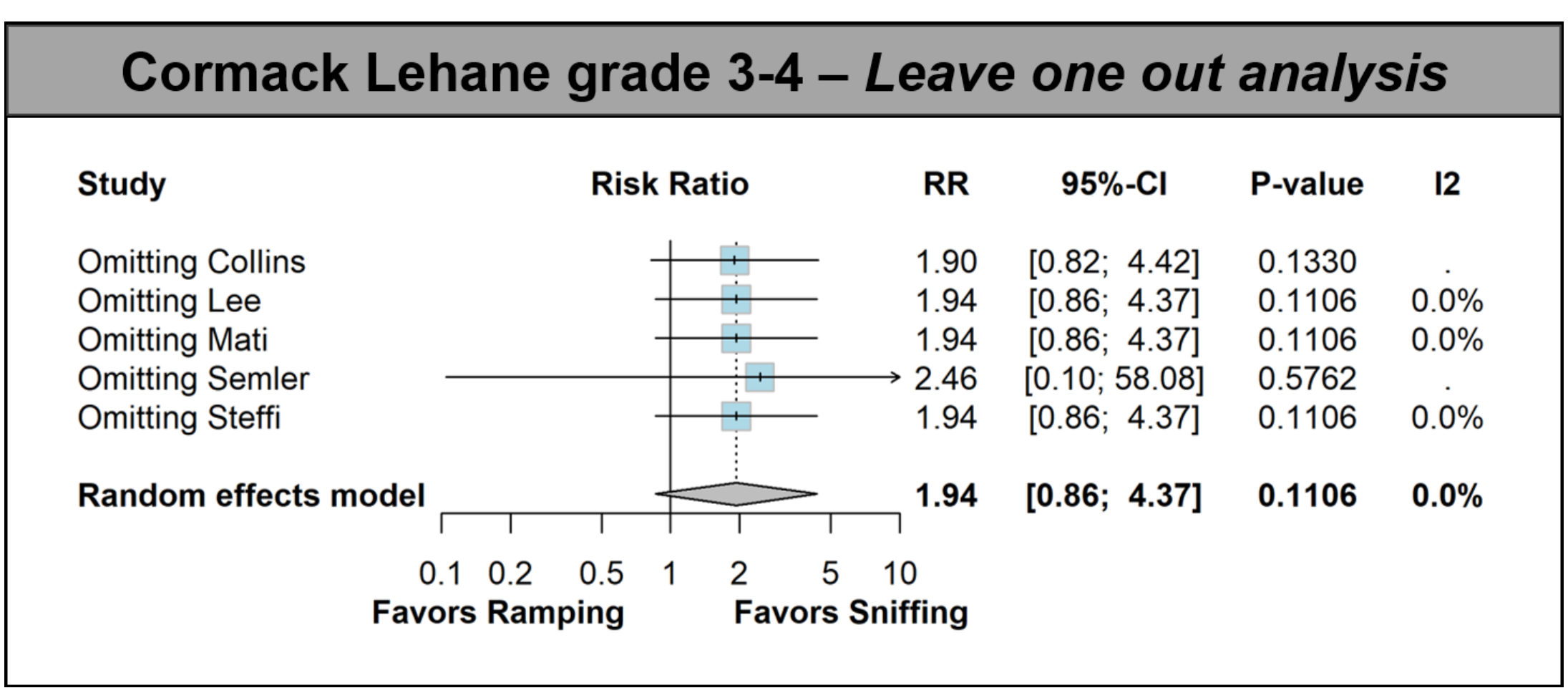

Figure 2SB - Leave one out sensitivity analysis of Cormack-Lehane grade 3 - 4.

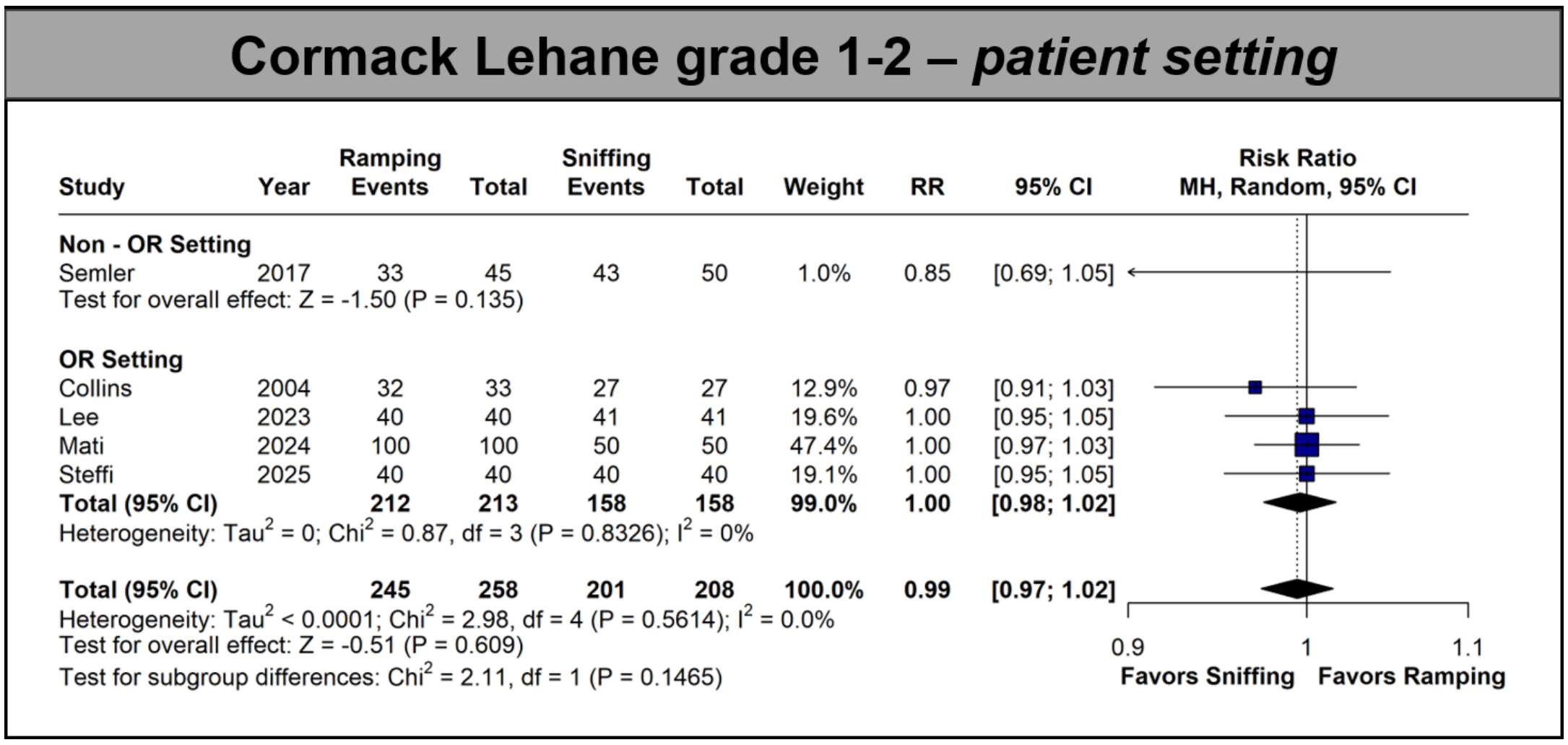

Figure 2SC - Random effects meta-analysis of Cormack-Lehane grade 1 - 2 stratified by patient setting.

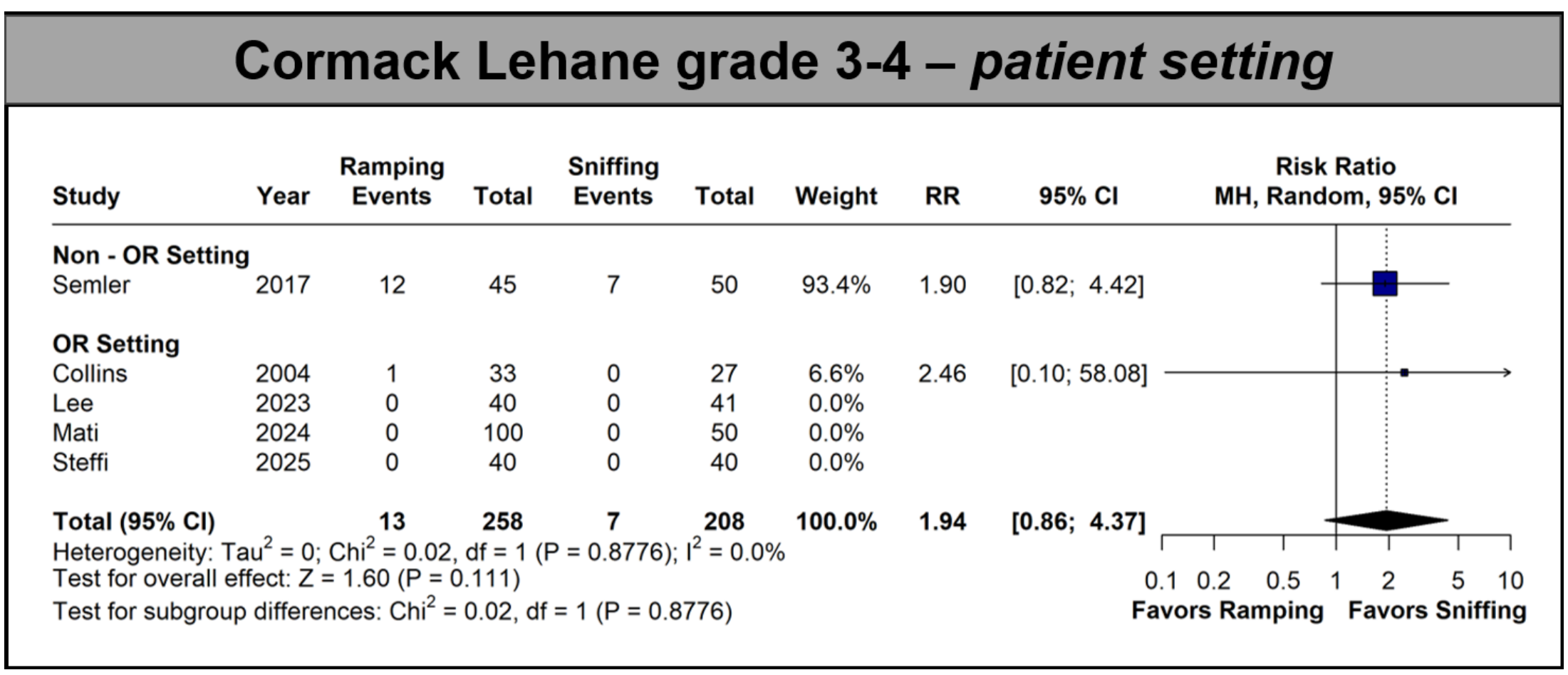

Figure 2SD - Random effects meta-analysis of Cormack-Lehane grade 3 - 4 stratified by patient setting.

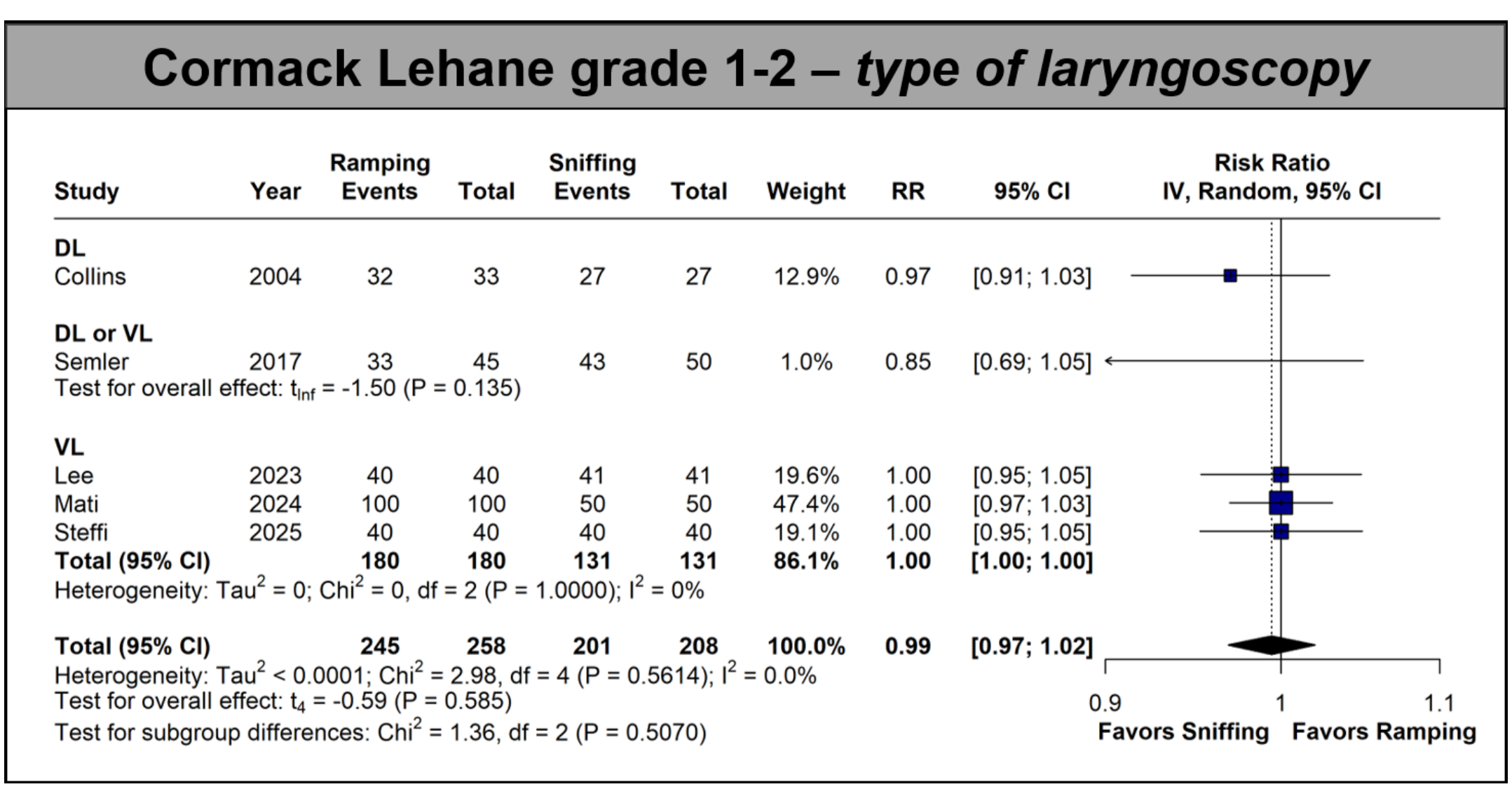

Figure 2SE - Random effects meta-analysis of Cormack-Lehane grade 1 - 2 stratified by type of laryngoscopy.

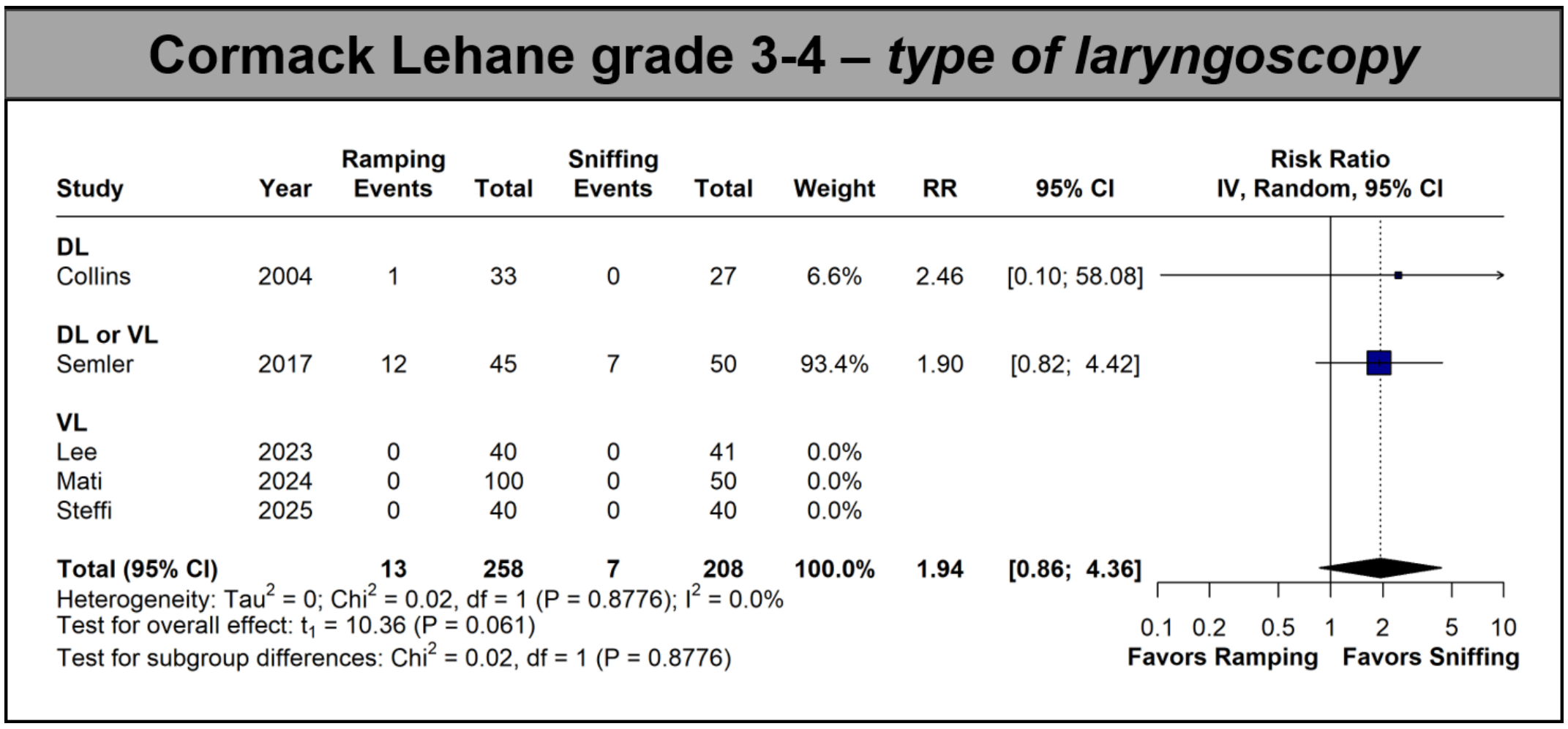

Figure 2SF - Random effects meta-analysis of Cormack-Lehane grade 3 - 4 stratified by type of laryngoscopy.

POST-HOC ANALYSIS OF PRIMARY OUTCOMES INCORPORATING GENERIC INVERSE VARIANCE AND HARTUNG-KNAPP-SIDIK-JONKMAN STATISTICAL METHODS

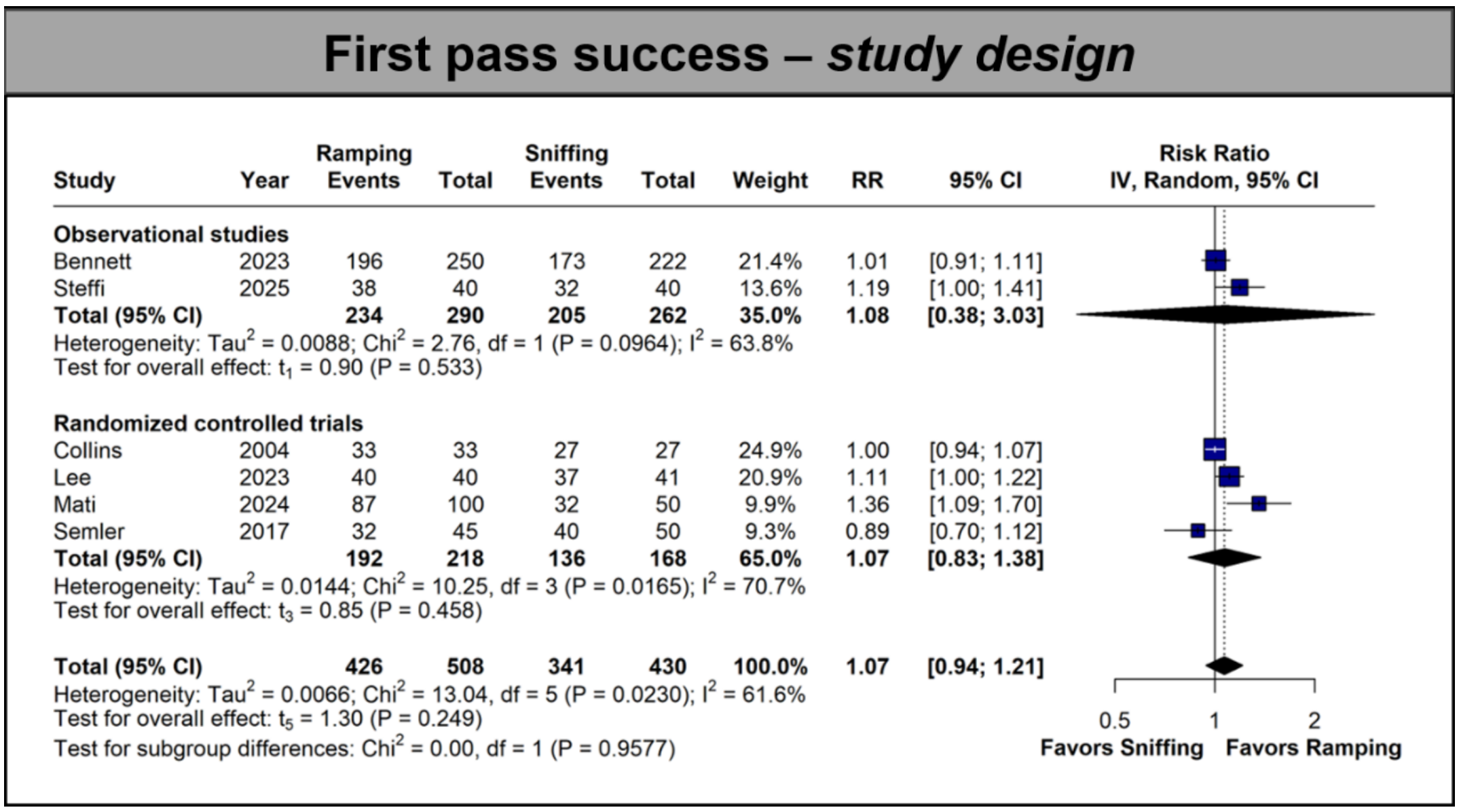

Figure 3SA - Random effects meta-analysis of first pass success incorporating Generic Inverse Variance and Hartung-Knapp-Sidik-Jonkman method stratified by study design.

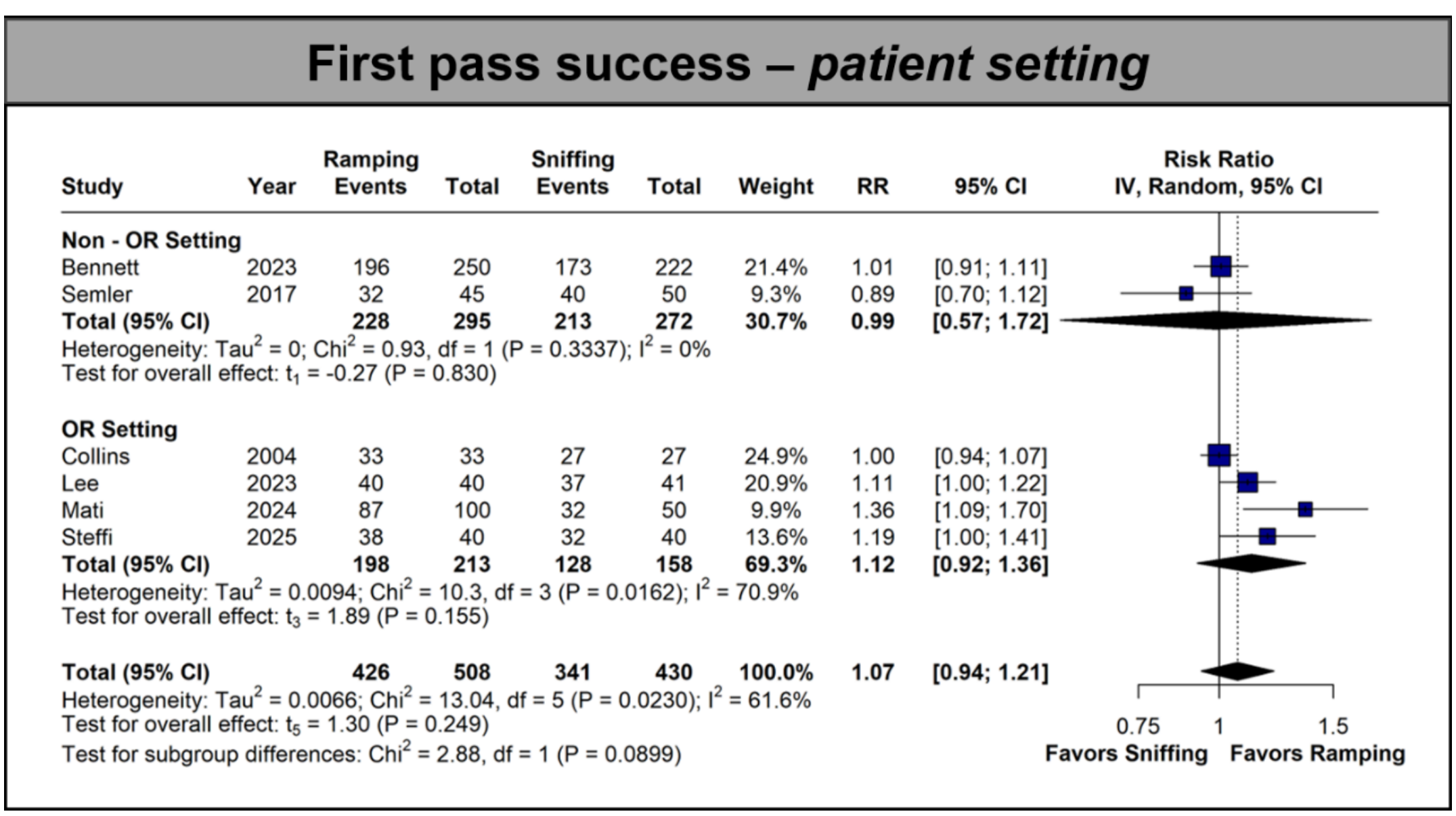

Figure 3SB - Random effects meta-analysis of first pass success incorporating Generic Inverse Variance and Hartung-Knapp-Sidik-Jonkman method stratified by patient setting.

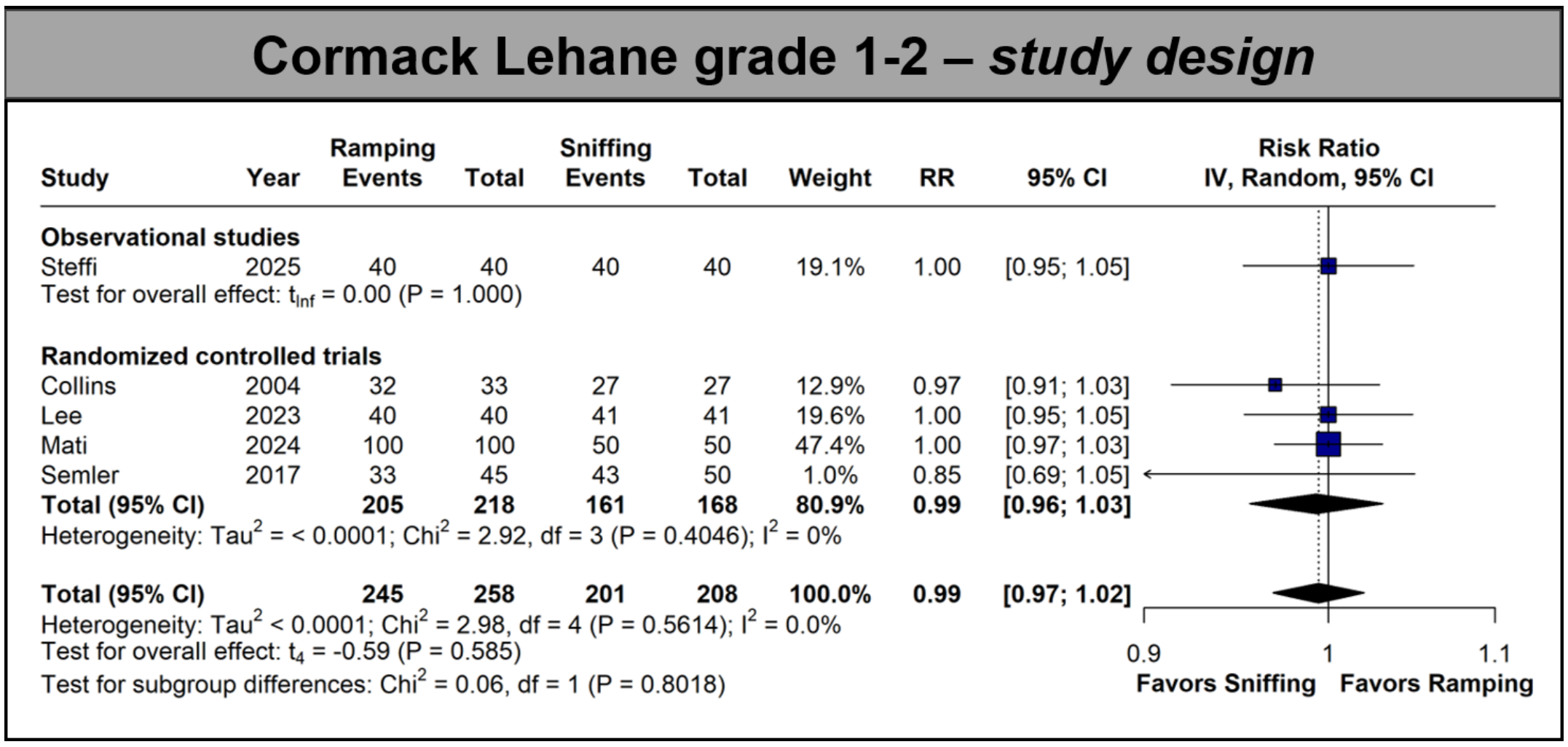

Figure 3SC - Random effects meta-analysis of Cormack-Lehane grade 1 - 2 incorporating Generic Inverse Variance and Hartung-Knapp-Sidik-Jonkman method stratified by study design.

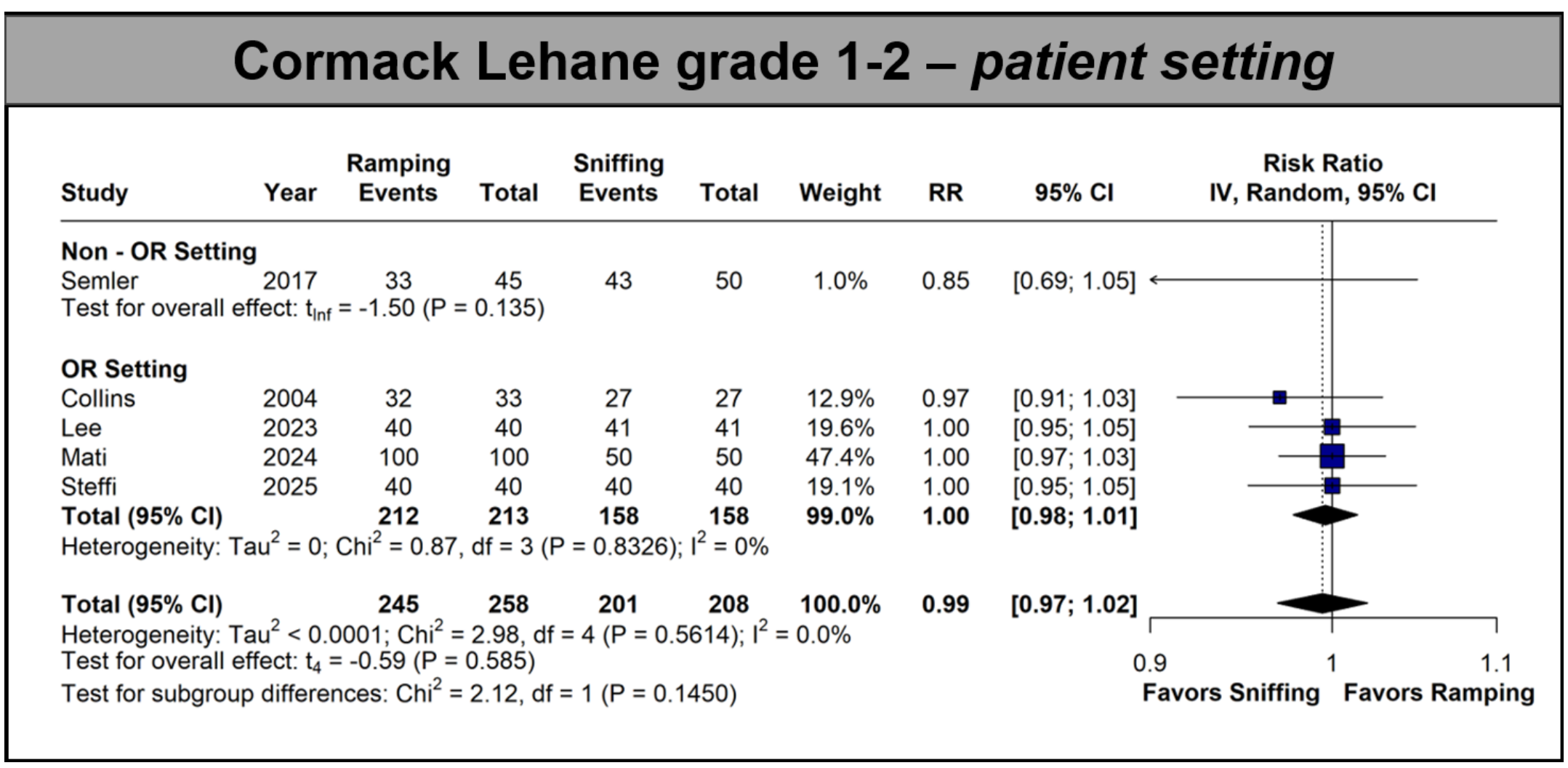

Figure 3SD - Random effects meta-analysis of Cormack-Lehane grade 1 - 2 incorporating Generic Inverse Variance and Hartung-Knapp-Sidik-Jonkman method stratified by patient setting.

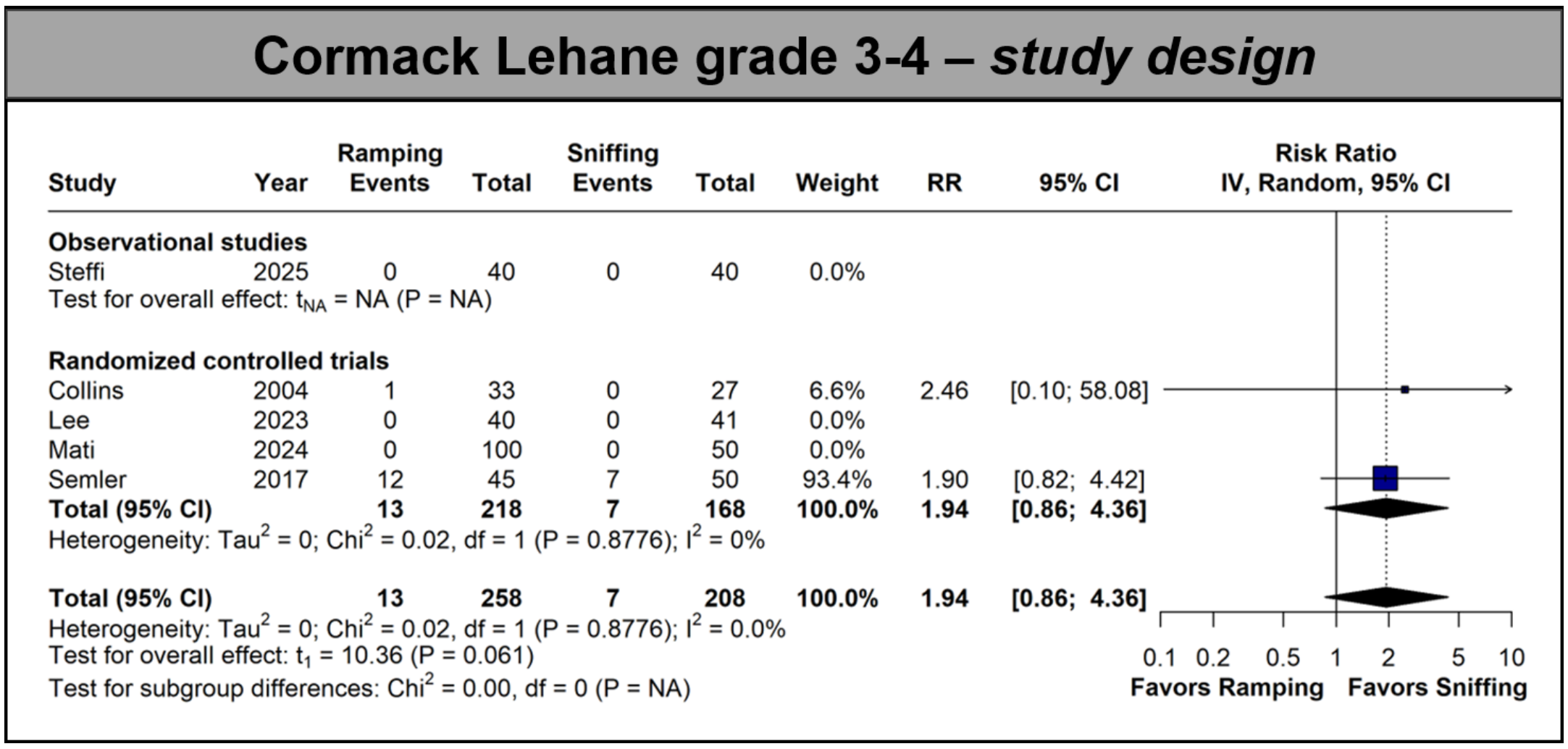

Figure 3SE - Random effects meta-analysis of Cormack-Lehane grade 3 - 4 incorporating Generic Inverse Variance and Hartung-Knapp-Sidik-Jonkman method stratified by study design.

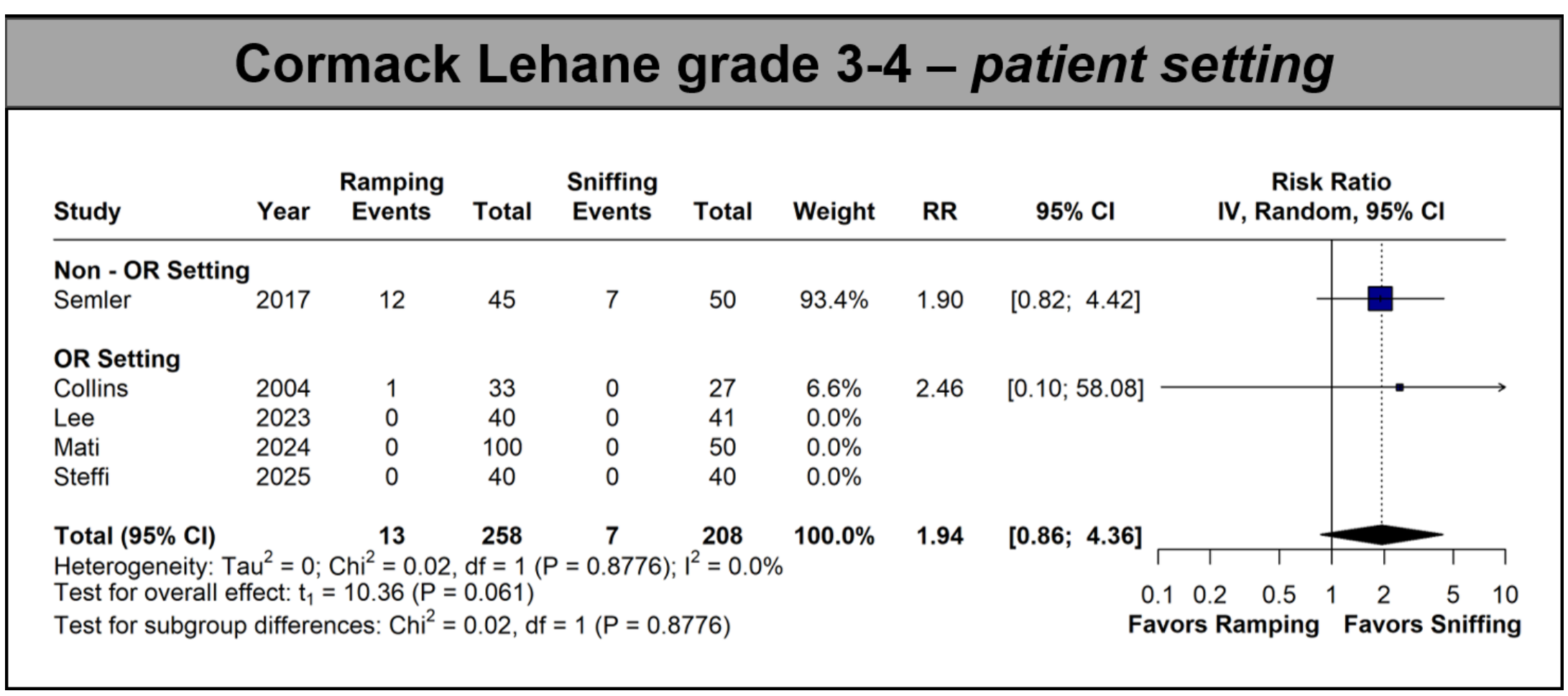

Figure 3SF - Random effects meta-analysis of Cormack-Lehane grade 3 - 4 incorporating Generic Inverse Variance and Hartung-Knapp-Sidik-Jonkman method stratified by patient setting.

SECONDARY OUTCOMES

Intubations requiring > 1 attempt

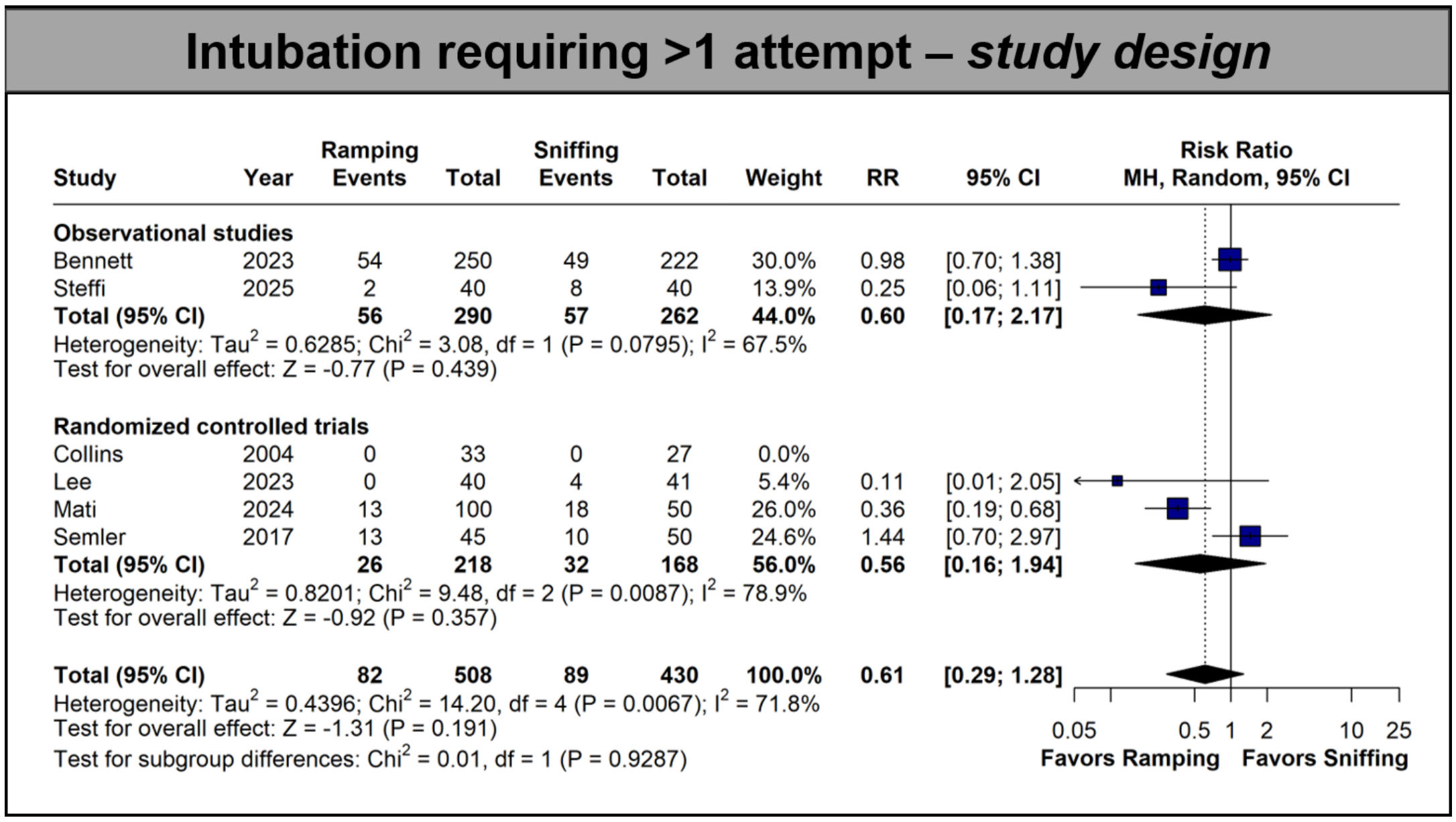

Figure 4SA - Random effects meta-analysis of intubations requiring > 1 attempt stratified by study design.

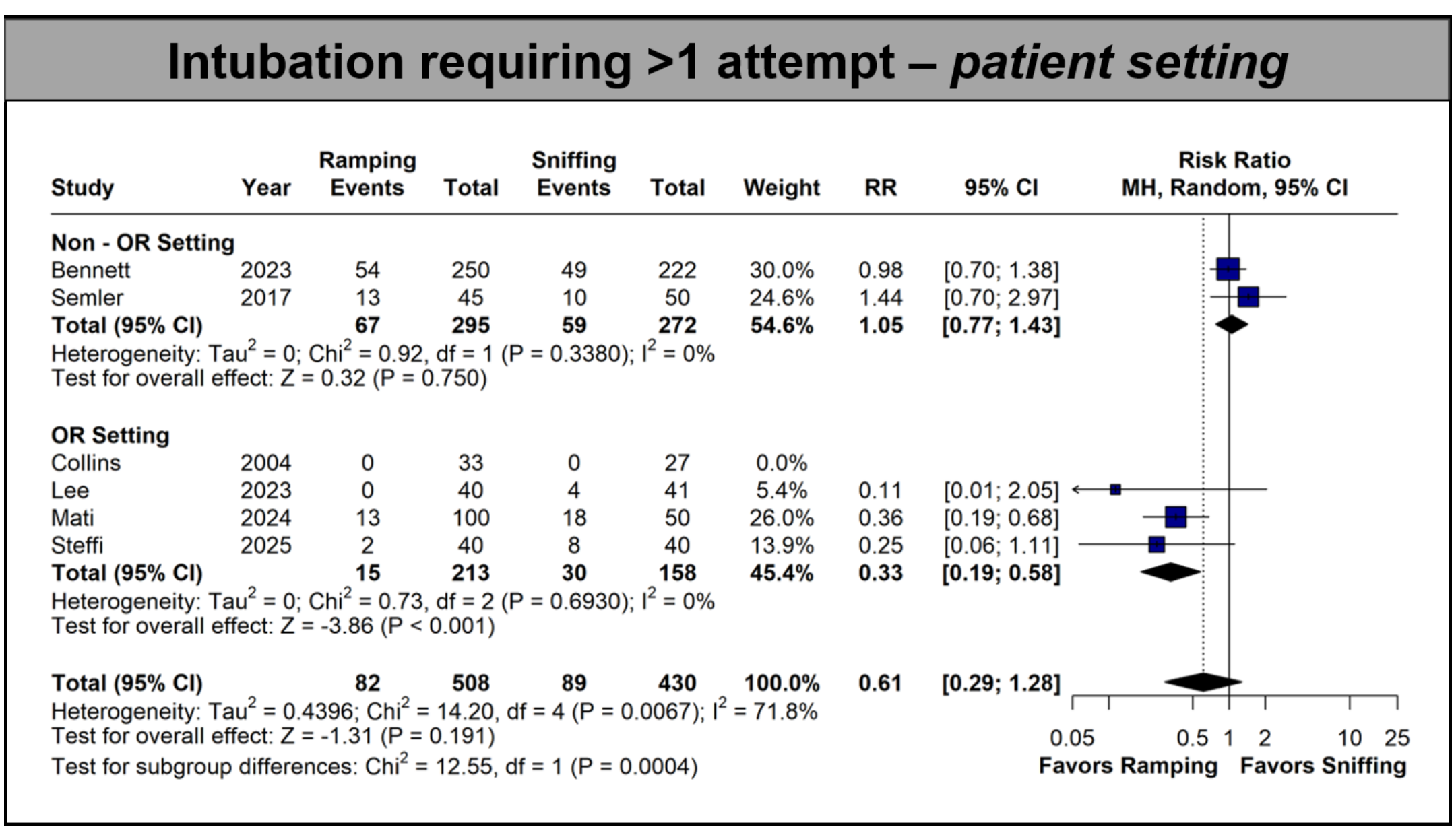

Figure 4SB - Random effects meta-analysis of intubations requiring > 1 attempt stratified by patient setting.

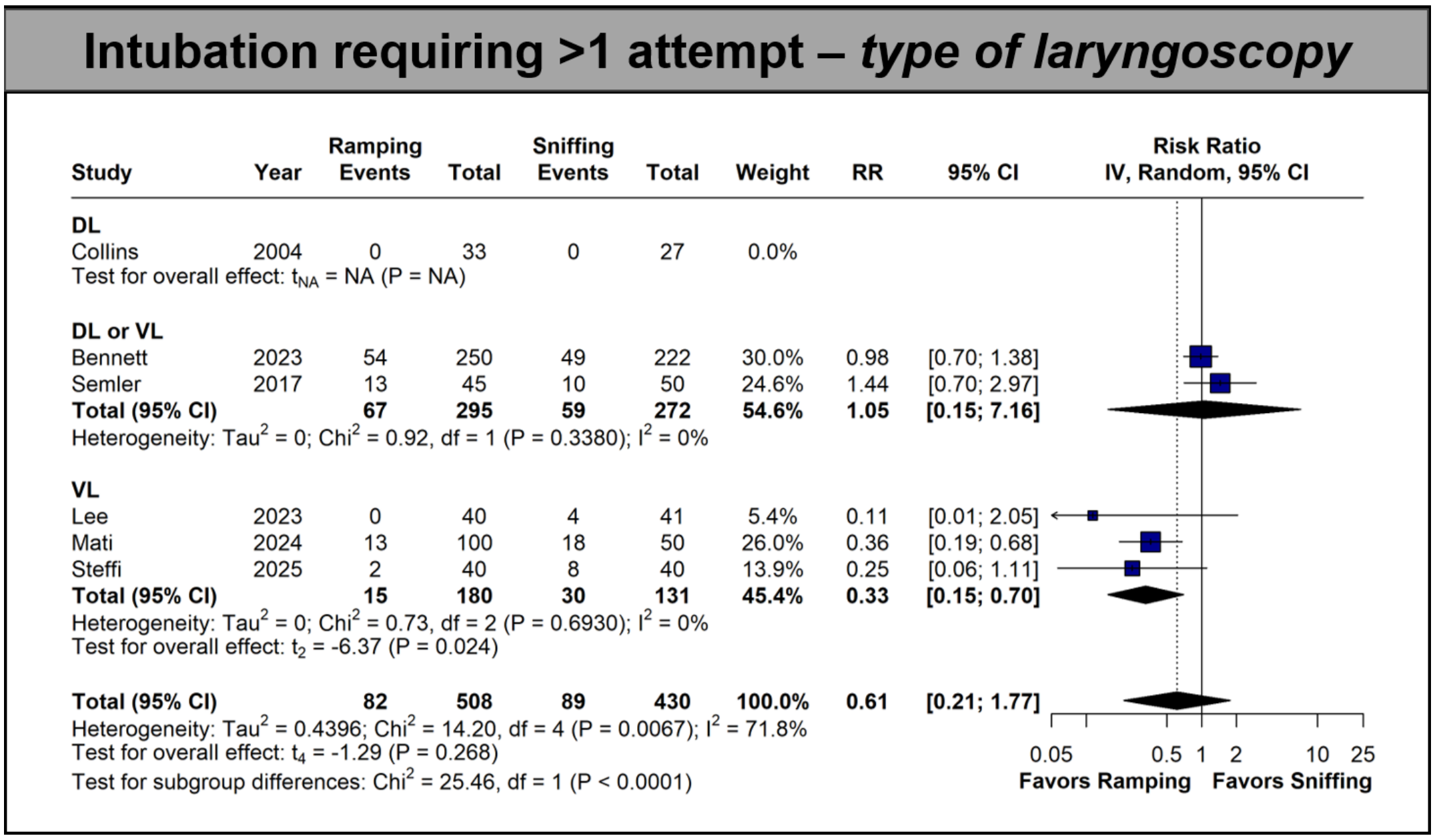

Figure 4SC - Random effects meta-analysis of intubations requiring > 1 attempt stratified by type of laryngoscopy.

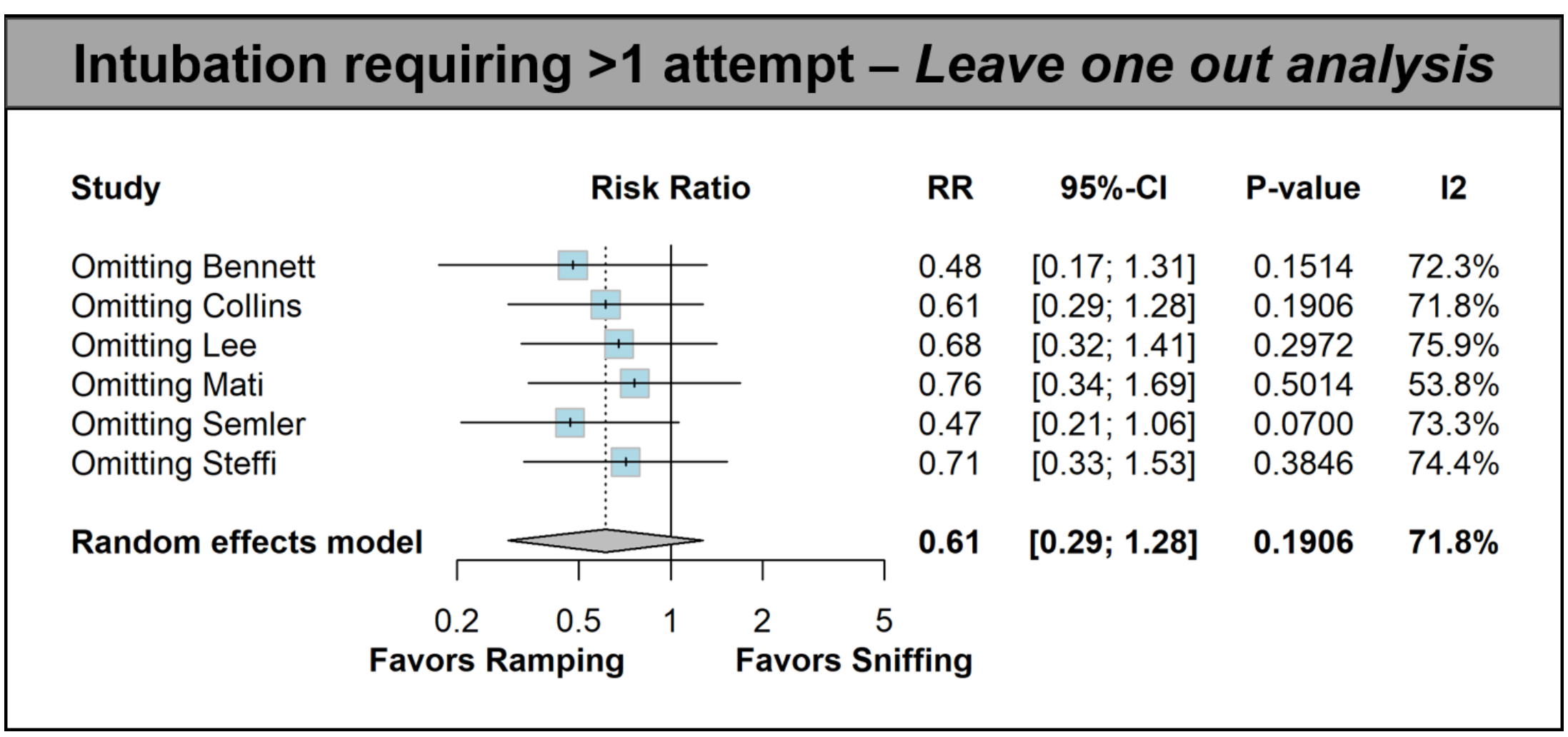

Figure 4SD - Leave one out sensitivity analysis of intubations requiring > 1 attempt.

Mean laryngoscopy time

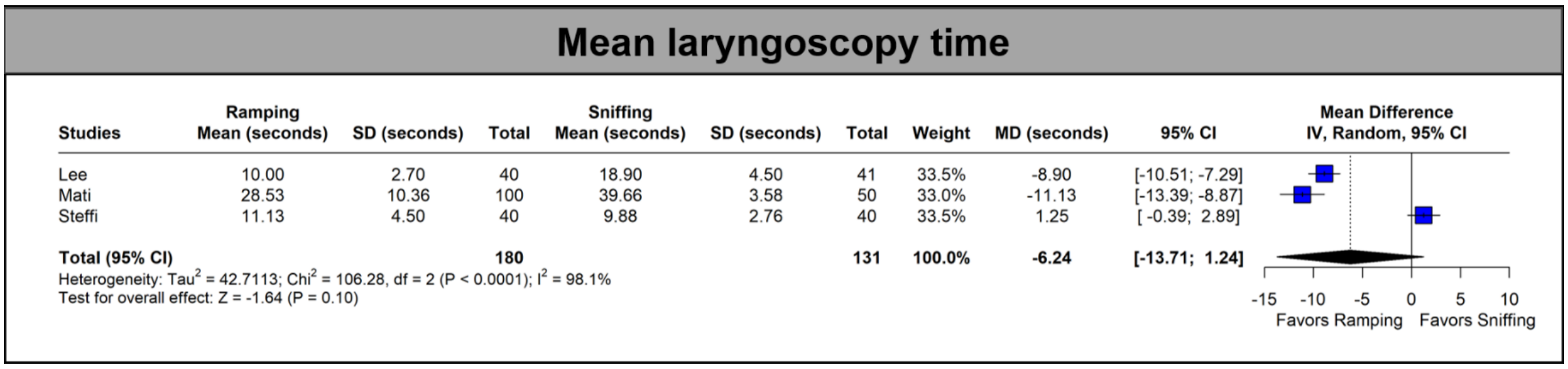

Figure 5SA - Random effects meta-analysis of mean laryngoscopy time.

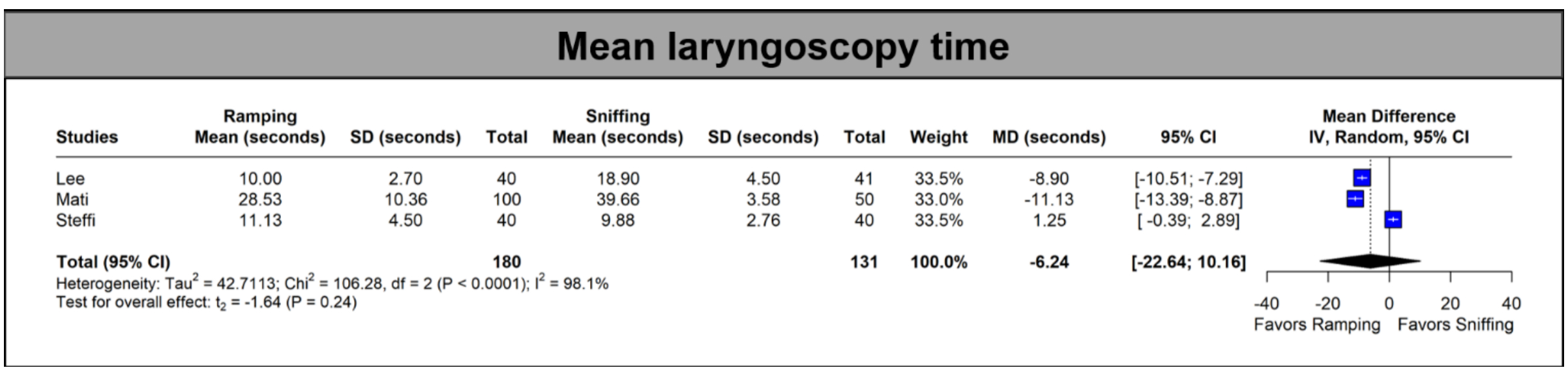

Figure 5SB - Random effects meta-analysis of mean laryngoscopy time incorporating Hartung-Knapp-Sidik-Jonkman method.

Mean tube insertion time

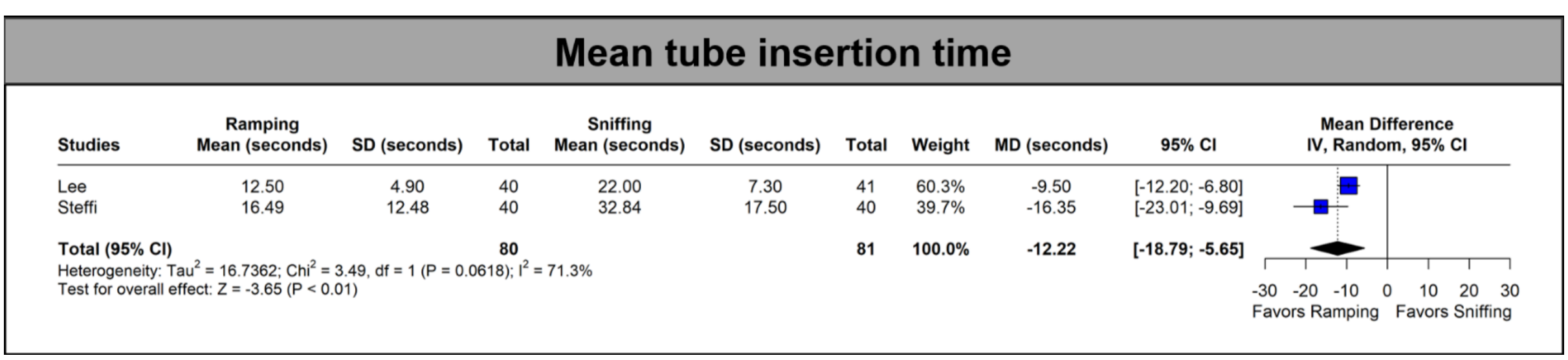

Figure 5SC - Random effects meta-analysis of mean tube insertion time.

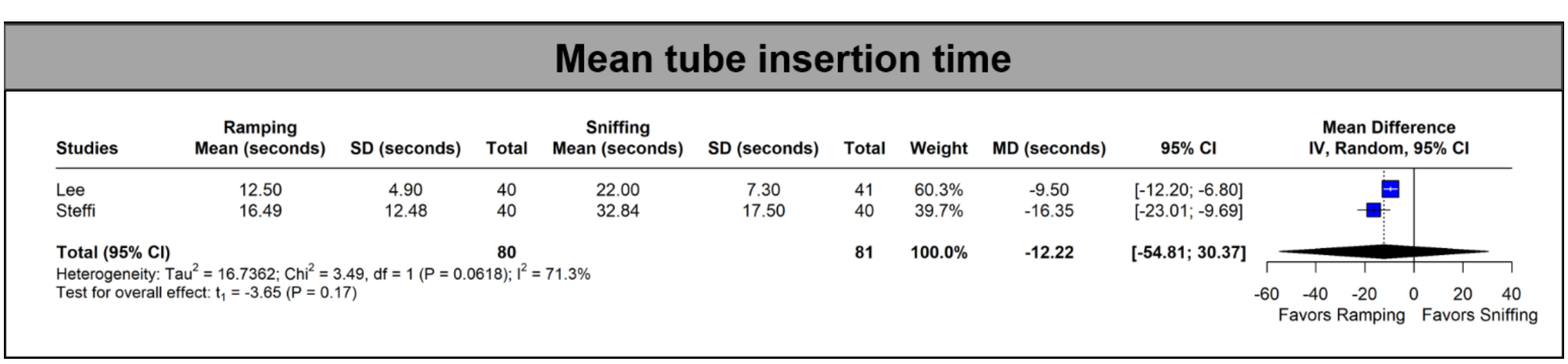

Figure 5SD - Random effects meta-analysis of mean tube insertion time incorporating Hartung-Knapp-Sidik-Jonkman method.

Mean intubation time

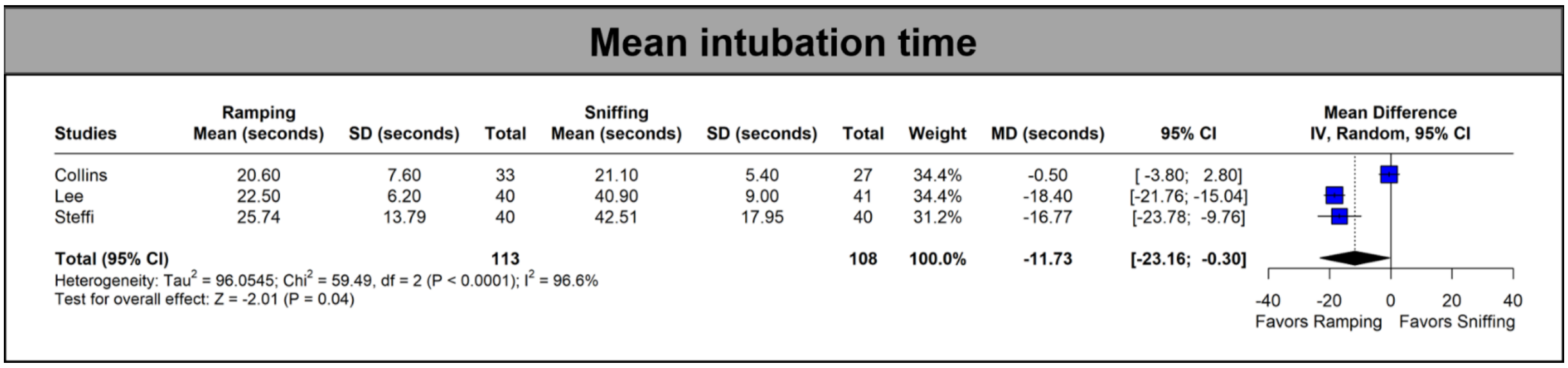

Figure 5SE - Random effects meta-analysis of mean intubation time.

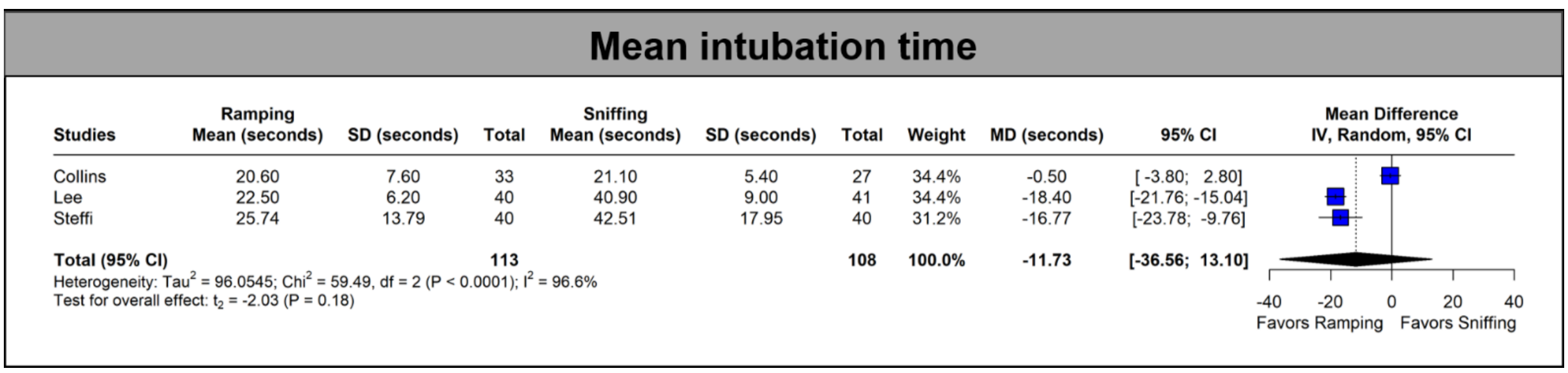

Figure 5SF - Random effects meta-analysis of mean intubation time incorporating Hartung-Knapp-Sidik-Jonkman method.

RISK OF BIAS ASSESSMENT

Risk of bias assessment of randomized studies

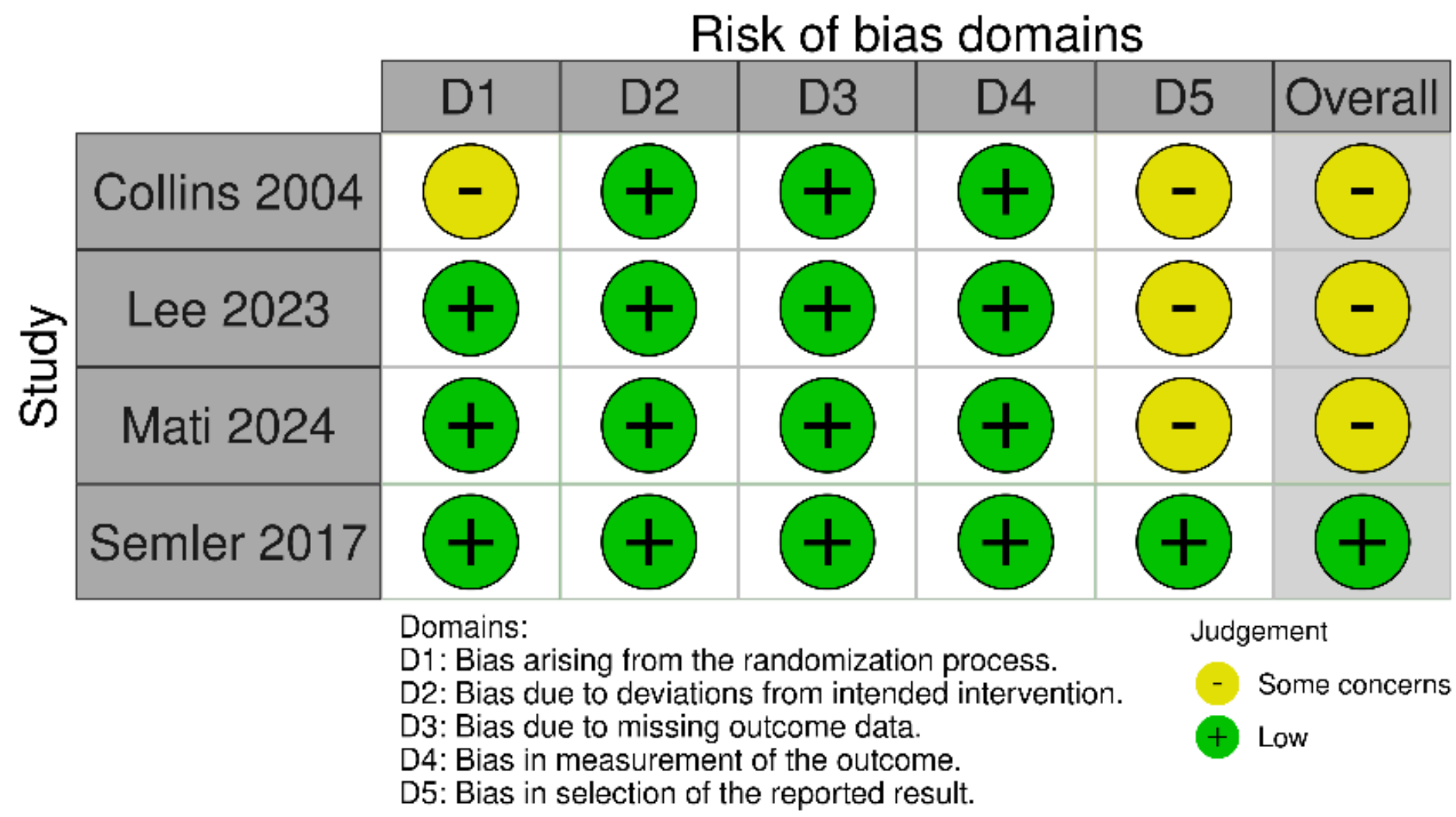

Figure 6SA - Risk of bias assessment of first pass success.

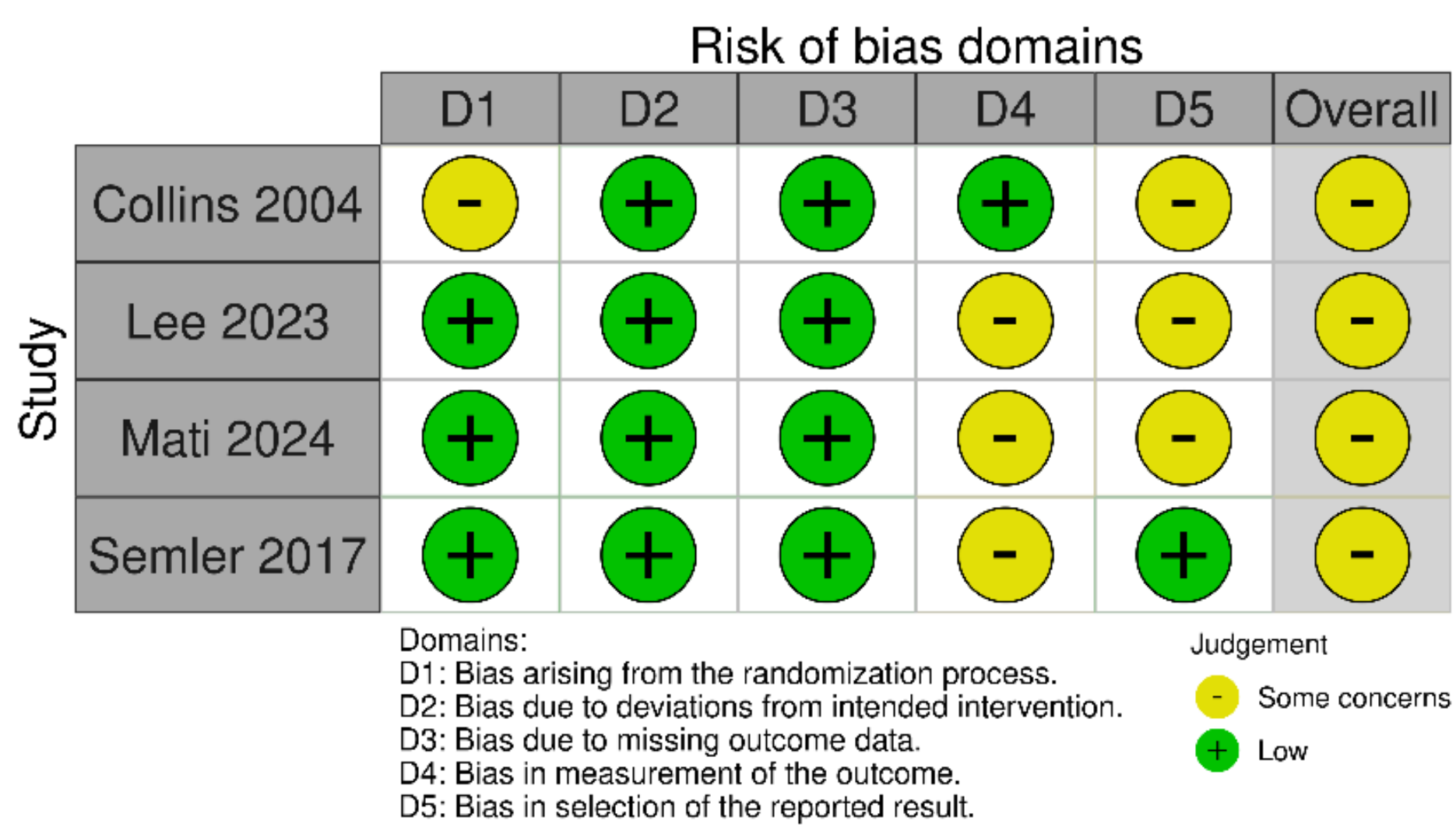

Figure 6SB - Risk of bias assessment of Cormack Lehane grading.

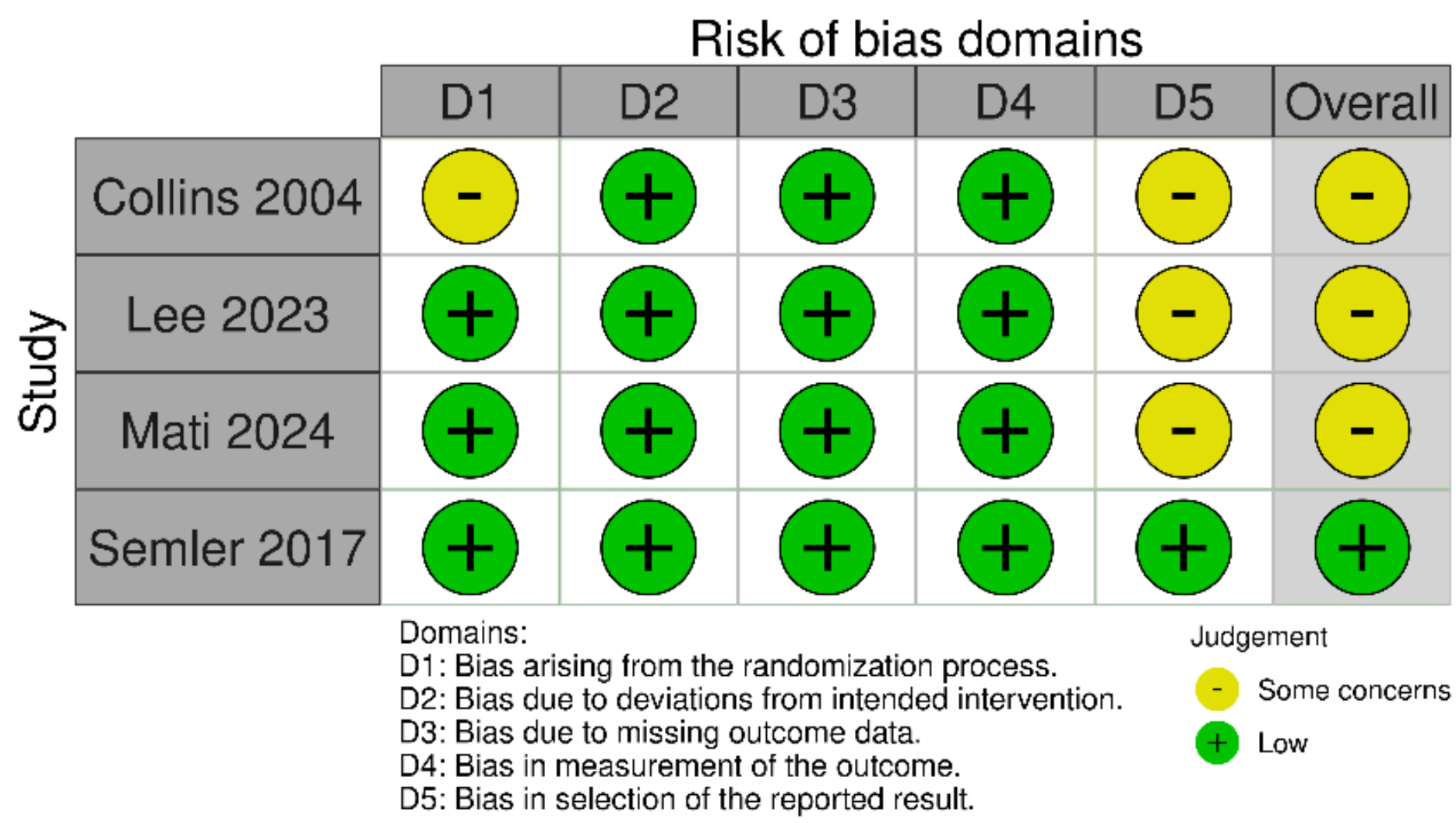

Figure 6SC - Risk of bias assessment of intubation requiring > 1 attempt.

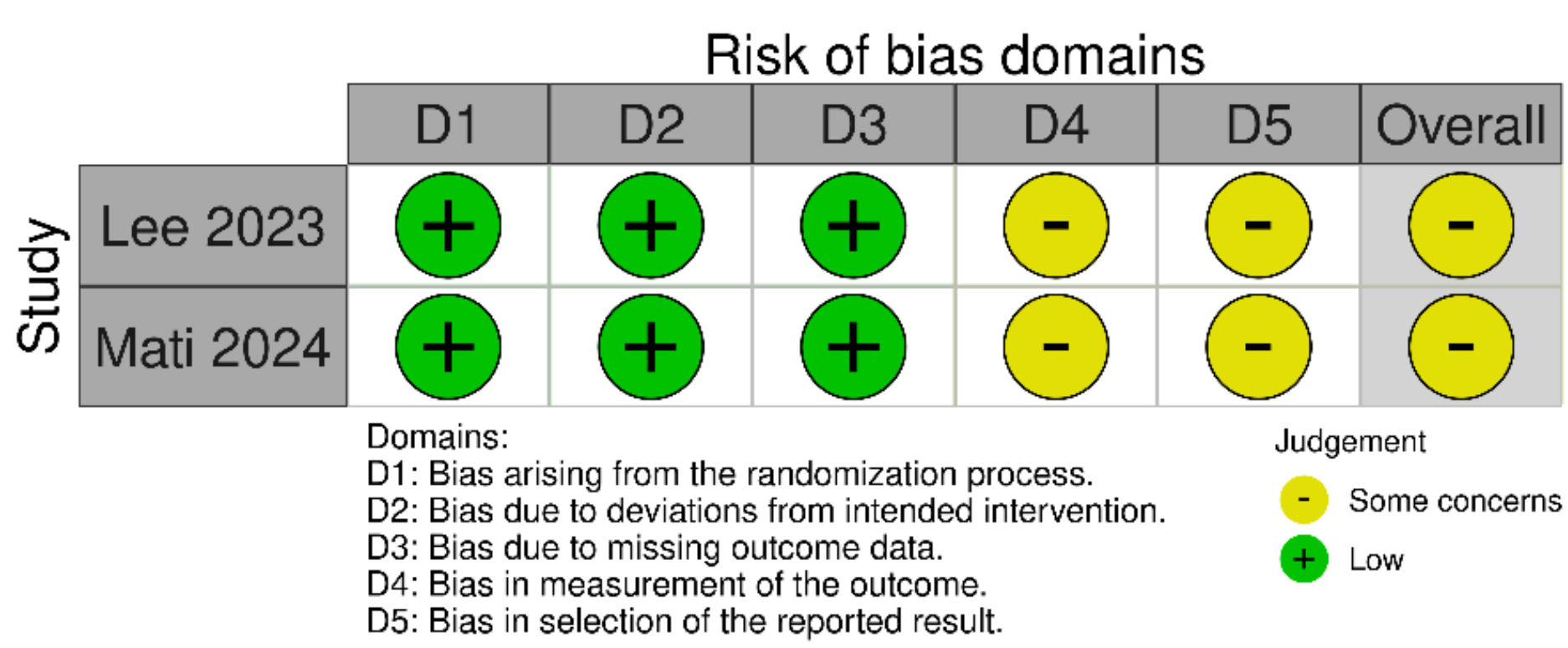

Figure 6SD - Risk of bias assessment of mean laryngoscopy time.

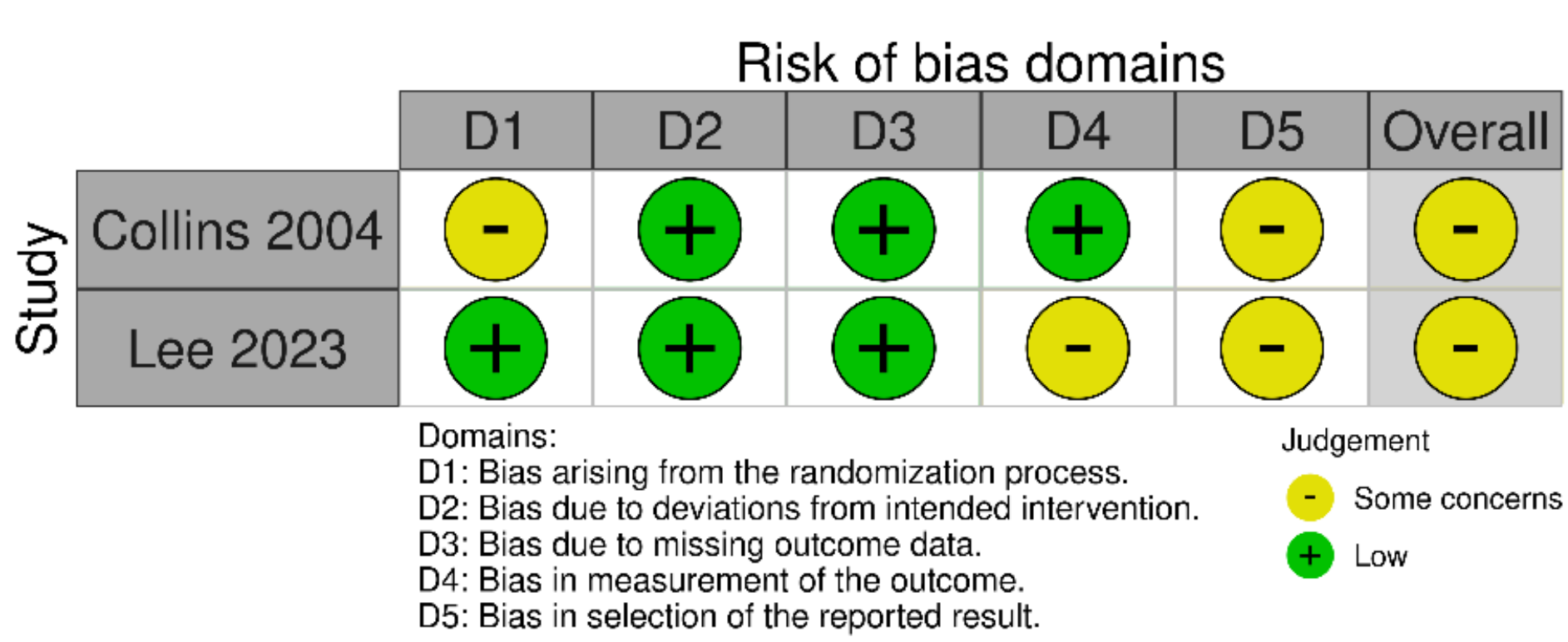

Figure 6SE - Risk of bias assessment of mean intubation time.

RISK OF BIAS ASSESSMENT OF NON-RANDOMIZED STUDIES

Target randomized trials for the studies included

Bennett 2023 et al. [3]

"Comparison of first pass success with head elevated position (ramped or pillow/occipital pillow or bed tilt) and flat positioning for endotracheal intubation in adults in the emergency department".

Participants and eligible criteria

- **Inclusion criteria**

Patients of age > 16 years undergoing endotracheal intubation in the emergency department.

- **Exclusion criteria**

Patients will be excluded if they (1) require surgical airway, awake fibreoptic intubation, emergency cricothyroid intubation, prehospital intubation; (2) are diagnosed with congenital syndromes; (3) unknown weight status; (4) are declared dead on arrival.

Treatment strategies

Intervention A - Endotracheal intubation in the ramped or head up position by elevation of the head and shoulders to achieve horizontal alignment of the external auditory meatus and sternal notch.

Intervention B - Endotracheal intubation with pillow or occipital pad by flexion of the neck and extension of the head.

Intervention C - Endotracheal intubation with bed tilt position by elevating head end of bed by 20-30 degrees.

Control - Endotracheal intubation in flat position by laying the patient supine on the bed.

Assignment procedures

Randomization of eligible patients to one of four groups with computer generated random sequence without blinding.

Follow-up

Follow-up will begin at the time of assignment to a treatment strategy. Follow up will end earliest of discharge from the ED or death.

Outcomes

1. Primary outcome:
    a. First pass success defined as successful endotracheal intubation on the first attempt.
2. Secondary outcome:
    a. Any adverse event including oxygen desaturation, bradycardia, tachycardia, hypotension, hypertension, cardiac arrest, aspiration, laryngospasm, mainstem bronchus intubation, esophageal intubation, airway trauma, dental trauma, equipment failure, or medication error.
    b. Difficulty intubation dichotomized as difficult (Cormack Lehane grade 3 or 4) or easy (Cormack Lehane grade 1 or 2).

Causal contrasts

Intention-to-treat effect.

Steffi 2025 et al.[15]

"The Impact of Patient Positioning on Video Laryngoscopy-Guided Tracheal Intubation in Obese Patients: A Prospective Observational Study Comparing Ramped versus Sniffing Position".

Participants and eligible criteria

– **Inclusion criteria**

Patients of age 18 - 80 years with BMI greater than 30kg/m^2 undergoing elective surgery requiring endotracheal intubation under general anesthesia.

– **Exclusion criteria**

Patients will be excluded if they (1) have a known history of difficult intubation, cervical spine pathology, previous head or neck surgery, increased risk of aspiration; (2) require rapid sequence intubation; (3) have an anticipated difficult airway based on the American Society of Anesthesiologists' (ASA) Difficult Airway Algorithm, the criterion being Mallampati class III-IV combined with limited mouth opening, thyromental distance less than 6 cm or restricted cervical spine mobility.

Treatment strategies

Intervention - Endotracheal intubation in the ramped position by elevation of the head and upper torso using blankets or cushions to achieve horizontal alignment of the external auditory meatus and sternal notch.

Control - Endotracheal intubation in the sniffing position by flexion of the neck and extension of the head using a 7cm pillow under the occiput.

Assignment procedures

Randomization of eligible patients to the intervention or control group with computer generated random sequence without blinding.

Follow-up

Follow-up will begin at the time of assignment to a treatment strategy. Follow up will end at the following extubating after completion of surgery or death, whichever occurs earlier.

Outcomes

1. Primary outcome:
    a. Total intubation time defined as the sum of laryngoscopy time and endotracheal tube insertion time. Laryngoscopy time is the interval between the insertion of the laryngoscope blade between the lips and attainment of the best glottic view. Endotracheal tube insertion time is the interval from introduction of the tube into the oral cavity until its passage through the vocal cords.
2. Secondary outcome:
    a. Cormack-Lehane grading of laryngeal view.
    b. Number of intubation attempts.

c. Intubation success defined intubation completion within 90 seconds, oxygen saturation greater than 92%, and requiring no more than two attempts.

d. Adverse events including oxygen desaturation below 92%, mucosal trauma, esophageal intubation and dental injury.

Causal contrasts

Per-protocol analysis.

RISK OF BIAS ASSESSMENT OF NON-RANDOMIZED STUDIES

Study	D1	D2	D3	D4	D5	D6	Overall
Bennett 2023	Serious	Low	Low	Serious	Low	Moderate	Serious
Steffi 2025	Serious	Low	Low	Low	Low	Moderate	Serious

D1 Bias due to confounding
D2 Bias in classification on interventions
D3 Bias in selection of participants
D4 Bias due to missing data
D5 Bias in measurement of outcome
D6 Bias in selection of reported result

Figure 6SF - Risk of bias assessment of first pass success.

Study	D1	D2	D3	D4	D5	D6	Overall
Steffi 2025	Serious	Low	Low	Low	Moderate	Moderate	Serious

D1 Bias due to confounding
D2 Bias in classification on interventions
D3 Bias in selection of participants
D4 Bias due to missing data
D5 Bias in measurement of outcome
D6 Bias in selection of reported result

Figure 6SG - Risk of bias assessment of Cormack Lehane grading.

Study	D1	D2	D3	D4	D5	D6	Overall
Bennett 2023	Serious	Low	Low	Serious	Low	Moderate	Serious
Steffi 2025	Serious	Low	Low	Low	Low	Moderate	Serious

D1 Bias due to confounding
D2 Bias in classification on interventions
D3 Bias in selection of participants
D4 Bias due to missing data
D5 Bias in measurement of outcome
D6 Bias in selection of reported result

Figure 6SH - Risk of bias assessment of intubation requiring > 1 attempt.

Study	D1	D2	D3	D4	D5	D6	Overall
Steffi 2025	Serious	Low	Low	Low	Moderate	Moderate	Serious

D1 Bias due to confounding
D2 Bias in classification on interventions
D3 Bias in selection of participants
D4 Bias due to missing data
D5 Bias in measurement of outcome
D6 Bias in selection of reported result

Figure 6SI - Risk of bias assessment of mean laryngoscopy time.

Study	D1	D2	D3	D4	D5	D6	Overall
Steffi 2025	Serious	Low	Low	Low	Moderate	Moderate	Serious

D1 Bias due to confounding
D2 Bias in classification on interventions
D3 Bias in selection of participants
D4 Bias due to missing data
D5 Bias in measurement of outcome
D6 Bias in selection of reported result

Figure 6SJ - Risk of bias assessment of mean tube insertion time.

Study	D1	D2	D3	D4	D5	D6	Overall
Steffi 2025	Serious	Low	Low	Low	Moderate	Moderate	Serious

D1 Bias due to confounding
D2 Bias in classification on interventions
D3 Bias in selection of participants
D4 Bias due to missing data
D5 Bias in measurement of outcome
D6 Bias in selection of reported result

Figure 6SK - Risk of bias assessment of mean intubation time.

Table 2S - Summary of findings and GRADE assessment of quality of evidence in randomized studies

Participants (studies) Follow-up	Certainty assessment						Summary of findings				
	Risk of bias	Inconsistency	Indirectness	Imprecision	Publication bias	Overall certainty of evidence	Study event rates (%)		Relative effect (95%CI)	Anticipated absolute effects	
							With sniffing	With ramping		Risk with sniffing	Risk difference with ramping
First pass success											
386 (4 RCTs)	Serious*	Serious†	Not serious	Not serious	Publication bias strongly suspected‡	⊕◯◯◯ Very low*†‡	136/168 (81.0)	192/218 (88.1)	RR 1.07 (0.93 to 1.23)	136/168 (81.0%)	57 more per 1,000 (from 57 fewer to 186 more)
Cormack Lehane grade 1 - 2											
386 (4 RCTs)	Serious§	Not serious	Not serious	Not serious	Publication bias strongly suspected‡	⊕⊕◯◯ Low‡§	161/168 (95.8)	205/218 (94.0)	RR 0.99 (0.97 to 1.02)	161/168 (95.8%)	10 fewer per 1,000 (from 29 fewer to 19 more)
Cormack Lehane grade 3 - 4											
386 (4 RCTs)	Serious§	Not serious	Not serious	Serious‡	Publication bias strongly suspected‡	⊕◯◯◯ Very low‡§¶	7/168 (4.2)	13/218 (6.0)	RR 1.94 (0.86 to 4.37)	7/168 (4.2%)	39 more per 1,000 (from 6 fewer to 140 more)
Intubation requiring > 1 attempt											
386 (4 RCTs)	Serious*	Serious||	Not serious	Serious#	Publication bias strongly suspected‡	⊕◯◯◯ Very low*‡||#	32/168 (19.0)	26/218 (11.9)	RR 0.56 (0.16 to 1.94)	32/168 (19.0%)	84 fewer per 1,000 (from 160 fewer to 179 more)
Mean laryngoscopy time											
231 (2 RCTs)	Serious§	Serious**	Serious††	Not serious	Publication bias strongly suspected‡	⊕◯◯◯ Very low‡§**††	91	140	-	91	MD 9.87 seconds lower (12.03 lower to 7.7 lower)
Mean tube insertion time											
81 (1 RCT)	Serious§	Not serious	Serious††	Not serious	Publication bias strongly suspected‡	⊕◯◯◯ Very low‡§††	41	40	-	41	MD 9.5 seconds lower (12.2 lower to 6.8 lower)
Mean intubation time											
141 (2 RCTs)	Serious§	Serious‡‡	Serious††	Serious§§	Publication bias strongly suspected‡	⊕◯◯◯ Very low‡§††‡‡§§	68	73	-	68	MD 9.45 seconds lower (26.99 lower to 8.09 higher)

95%CI - confidence interval; RCT - randomized controlled trial; MD - mean difference; RR - risk ratio. * Risk of bias assessed as 'some concerns' due to bias in selection of reported result because of unavailability of prior published protocol; † substantial heterogeneity unexplained by subgroup analysis (I2 = 70.7%); ‡ limited number of studies published in the literature (< 10); § risk of bias assessed as some concerns due to lack of blinding and unavailability of prior published protocol; ¶ wide confidence interval including both null effect and appreciable harm (95%CI 0.86 to 4.37); || substantial heterogeneity (I2 = 78.9%); # wide confidence interval including both null effect and appreciable harm/benefit (95%CI 0.16 to 1.94); ** substantial unexplained heterogeneity; †† pooled population contained only patients in the operating room; ‡‡ non overlapping confidence intervals with considerable heterogeneity; §§ wide confidence interval of effect estimate.

Table 3S - Summary of findings and GRADE assessment of quality of evidence in non-randomized studies

Participants (studies) Follow-up	Certainty assessment						Summary of findings				
	Risk of bias	Inconsistency	Indirectness	Imprecision	Publication bias	Overall certainty of evidence	Study event rates (%)		Relative effect (95%CI)	Anticipated absolute effects	
							With sniffing	With ramping		Risk with sniffing	Risk difference with ramping
First pass success											
552 (2 non-randomized studies)	Very serious*	Serious†	Not serious‡	Serious§	Publication bias strongly suspected¶	⊕○○○ Very low*†‡§¶	205/262 (78.2)	234/290 (80.7)	RR 1.08 (0.92 to 1.26)	205/262 (78.2%)	63 more per 1,000 (from 63 fewer to 203 more)
Intubation requiring >1 attempt											
552 (2 non-randomized studies)	Very serious*	Serious||	Not serious‡	Serious§	Publication bias strongly suspected¶	⊕○○○ Very low*‡§¶||	57/262 (21.8)	56/290 (19.3)	RR 0.60 (0.17 to 2.17)	57/262 (21.8%)	87 fewer per 1,000 (from 181 fewer to 255 more)
Cormack Lehane grade 1 - 2											
80 (1 non-randomized study)	Serious#	Not serious	Serious‡	Not serious	Publication bias strongly suspected¶	⊕○○○ Very low‡¶#	40/40 (100.0)	40/40 (100.0)	RR 1.00 (0.95 to 1.05)	40/40 (100.0%)	0 fewer per 1,000 (from 50 fewer to 50 more)
Cormack Lehane grade 3 - 4											
80 (1 non-randomized study)	Serious#	Not serious	Serious‡	Not serious	Publication bias strongly suspected¶	⊕○○○ Very low‡¶#	0/40 (0.0)	0/40 (0.0)	Not estimable	0/40 (0.0%)	
Mean laryngoscopy time											
80 (1 non-randomized study)	Serious#	Not serious	Serious‡	Not serious	Publication bias strongly suspected¶	⊕○○○ Very low‡¶#	40	40	-	40	MD 1.25 seconds higher (0.39 lower to 2.89 higher)
Mean tube insertion time											
80 (1 non-randomized study)	Serious#	Not serious	Serious‡	Not serious	Publication bias strongly suspected¶	⊕○○○ Very low‡¶#	40	40	-	40	MD 16.35 seconds lower (23.01 lower to 9.69 lower)
Mean intubation time											
80 (1 non-randomized study)	Serious#	Not serious	Serious‡	Not serious	Publication bias strongly suspected¶	⊕○○○ Very low‡¶·#	40	40	-	40	MD 16.77 seconds lower (23.78 lower to 9.76 lower)

CI - confidence interval; RR - risk ratio MD - mean difference.* Serious risk of bias due to unadjusted data and significant missing data; † substantial heterogeneity (I2 = 63.8%); ‡ population consists of patients in the operating room only; § wide confidence interval including null effect and appreciable harm/benefit; ¶ limited number of studies published in the literature (< 10); || substantial heterogeneity (I2 = 67.5%); # serious risk of bias due to unadjusted data and lack of blinding.

FUNNEL PLOTS FOR ASSESSMENT OF PUBLICATION BIAS FOR PRIMARY OUTCOMES

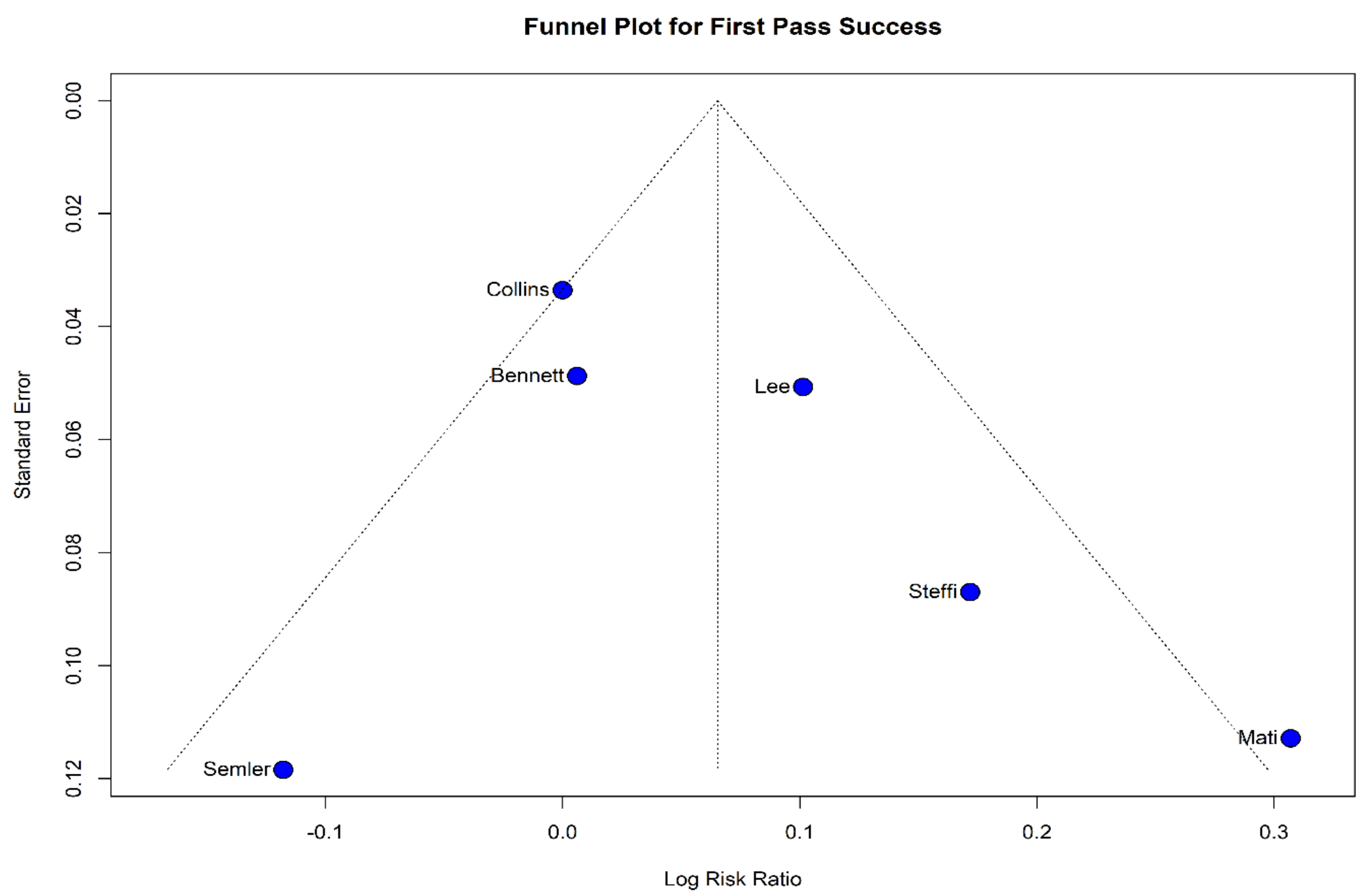

Figure 7SA - Funnel plot for first pass success.

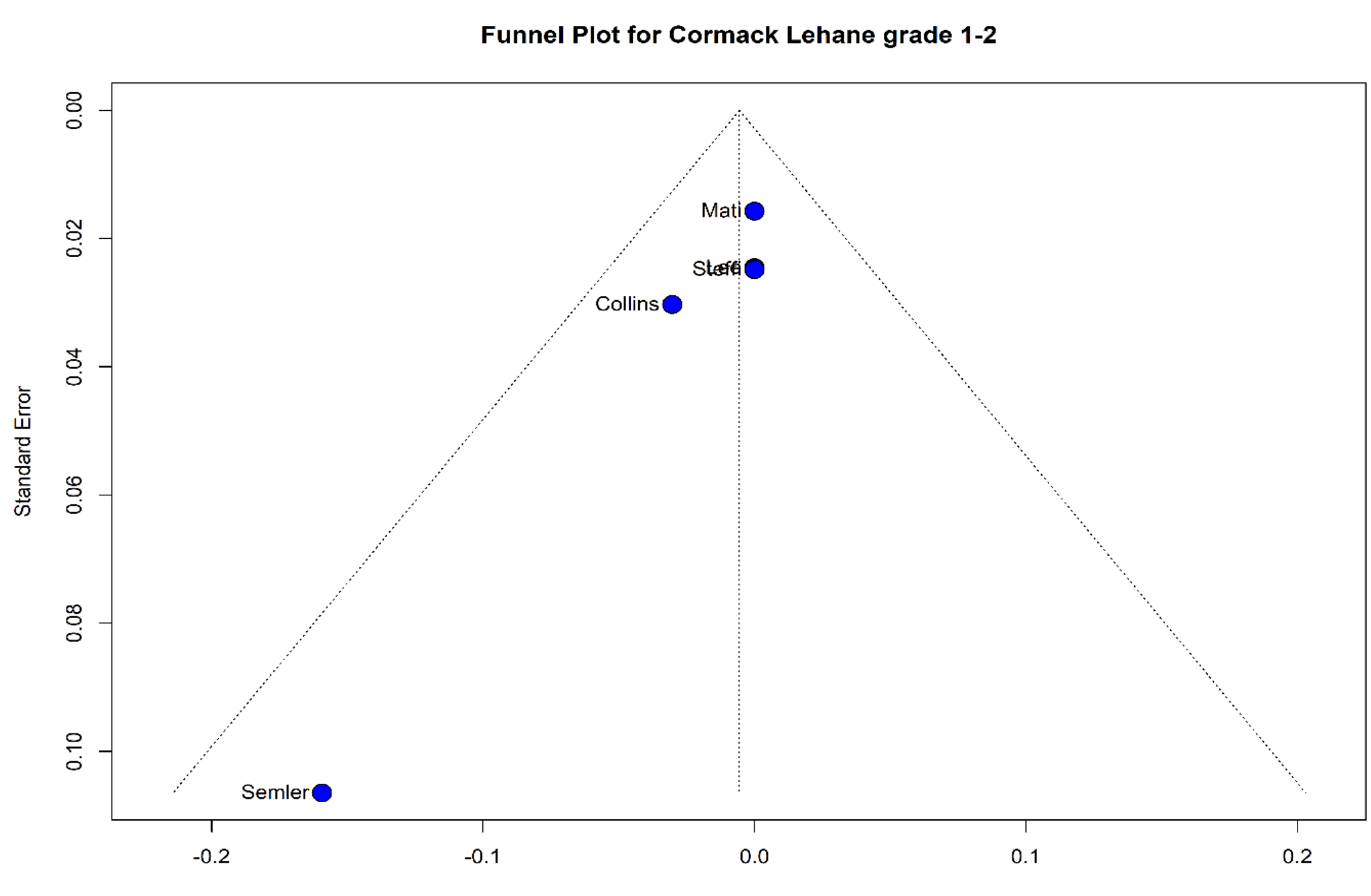

Figure 7SB - Funnel plot for Cormack Lehane grade 1 - 2.

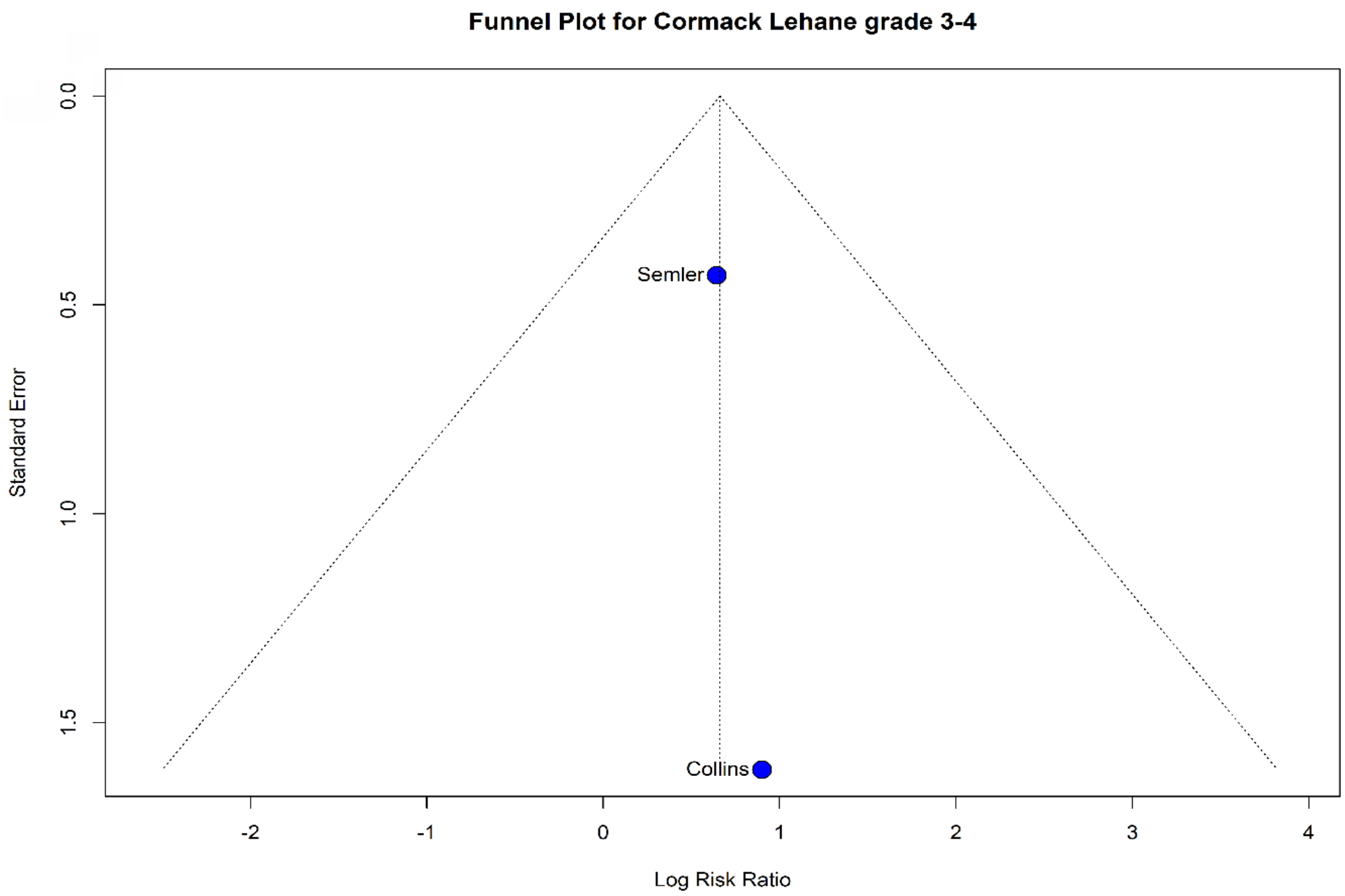

Figure 7SC - Funnel plot for Cormack Lehane grade 3 - 4.
